# Pathogenic SIV infection is associated with acceleration of epigenetic age in rhesus macaques

**DOI:** 10.1172/JCI189574

**Published:** 2025-07-15

**Authors:** Anna J. Jasinska, Ranjit Sivanandham, Sindhuja Sivanandham, Cuiling Xu, Juozas Gordevicius, Milda Milčiūtė, Robert T. Brooke, Paola Sette, Tianyu He, Egidio Brocca-Cofano, Benjamin B. Policicchio, Krishna Nayak, Saharsh Talwar, Haritha Annapureddy, Dongzhu Ma, Ruy M. Ribeiro, Cristian Apetrei, Ivona Pandrea

**Affiliations:** 1 Division of Infectious Diseases, Department of Medicine and; 2 Department of Pathology, School of Medicine, University of Pittsburgh, Pittsburgh, Pennsylvania, USA.; 3 Epigenetic Clock Development Foundation, Torrance, California, USA.; 4 Department of Infectious Diseases and Microbiology, Graduate School of Public Health, University of Pittsburgh, Pittsburgh, Pennsylvania, USA.; 5 Los Alamos National Laboratory (LANL), Los Alamos, New Mexico, USA.

**Keywords:** AIDS/HIV, Infectious disease, AIDS vaccine, Cardiovascular disease, Cellular senescence

## Abstract

HIV infection accelerates biological aging, but the contribution of the host’s age to this process is unknown. We investigated the influence of SIV infection in macaques (SIVmac) on the risk of comorbidities and aging in young and old rhesus macaques (RMs) by assessing pathogenesis markers, DNA methylation–based epigenetic age (EA), and EA acceleration (EAA) in blood and tissues. Initially, upon SIV infection, the young RMs showed greater resilience to CD4^+^ T cell depletion, better control of T cell activation, hypercoagulation, and excessive inflammation, yet this resilience was progressively lost in the advanced stages of infection. During the late stages of infection, the young RMs, but not the aged ones, showed an increase in EA in PBMCs; also, EAA in the cerebellum and heart of young RMs was higher compared with old RMs. SIV infection was more pathogenic in aged animals in early stages, leading to a more rapid disease progression; however, accelerated aging mostly affected young animals, so that the levels of multiple key pathogenesis markers in the young RMs converged toward those specific to aged ones in the late stages of infection. We conclude that SIV infection–driven age acceleration is tissue specific, and that host age influences the susceptibility of different tissues to enhanced aging.

## Abstract 2

We report here the first direct evidence that pathogenic SIV infection drives age acceleration in the RMs, thus offering a mechanistic explanation for HIV-associated comorbidities.

## Graphical abstract

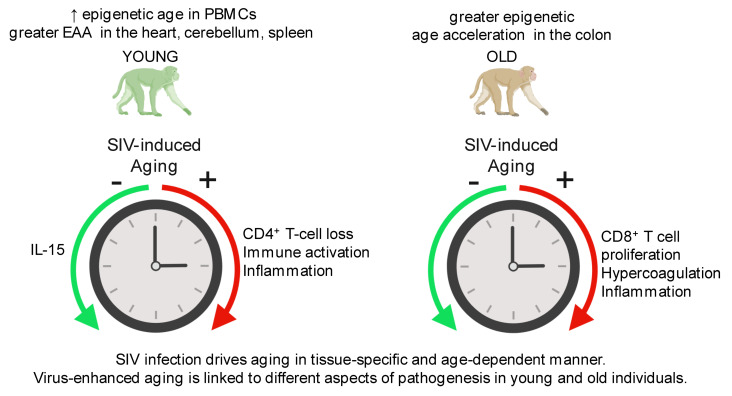

## Introduction

Antiretroviral therapy (ART) successfully suppresses HIV infection, dramatically increasing the life expectancy of people living with HIV (1). Currently, the life expectancy of a newly HIV-infected 20-year-old adult that initiates ART is 78 years (2). HIV infection has thus shifted from a deadly clinical condition to a lifelong chronic illness (1). Consequently, most people living with HIV are currently aged over 50 years (3), increased morbidity and mortality persist in elderly people living with HIV (4, 5), and current management of HIV infection focuses on the challenges faced by this group (1). Drivers of HIV pathogenicity that are not completely normalized by ART (i.e., chronic immune activation, inflammation, and microbial translocation) lead to HIV-associated non-AIDS conditions affecting multiple tissues and organs involved in immune, metabolic, and neurological processes (1, 4).

Natural aging is associated with stem cell exhaustion, altered intracellular communication, genomic instability, telomere attrition, epigenetic alterations, loss of protein homeostasis, altered growth factor sensing, mitochondrial dysfunction, and cellular senescence (6). Aging is associated with moderate increases of chronic inflammation, a hallmark of age-related diseases (7). Low levels of tissue infiltration with immune cells of both the innate and adaptive immune system persists in the elderly, leading to increased cytokine and chemokine levels (7, 8). Meanwhile, the continuous remodeling of lymphoid organs characteristic of immune senescence results in blunted immune responses (9). Furthermore, old age is associated with alteration of hemostasis regulation, leading to hypercoagulation and prothrombotic states (10, 11), with heightened risks for venous thrombosis, complicated or severe atherosclerosis, and pulmonary emboli (10), and may affect the long-term outcomes of diseases in the elderly (10, 11). Inflammation, T cell activation, and the hypercoagulable state are independent contributors to the risk of morbidity and mortality in people living with HIV (12–16); hence, physiological aging could render HIV-related pathologies more severe in elderly people living with HIV and increase the gravity of HIV-associated non-AIDS comorbidities.

Meanwhile, comorbidities occur earlier in people living with HIV than in age-matched uninfected individuals (17, 18), which led to the concept of premature/accelerated HIV-related senescence. There has been debate as to whether premature aging of people living with HIV is due to HIV, ART, or both. A direct proof that HIV causes premature aging is that telomeres are shorter in children infected with HIV than in controls (19), and thus the relative consensus is that premature aging of people living with HIV is due to HIV-related immunological senescence (20). However, ART contribution to accelerated aging in people living with HIV could not be discarded (21).

More recently, epigenetic clocks based on DNA methylation (DNAme), which are objective markers of biological aging and can capture the effects of exposures to aging-modulating factors (22), showed that HIV infection is associated with an increase in the DNAme-based epigenetic age (EA) in the circulation (5.2 years) and the brain (7.4 years) (23). A significant EA acceleration (EAA) occurs within 2–3 years after HIV infection (24, 25), before significant immunosuppression in ART-naive individuals with preserved immune status (26). Age acceleration in the brains of people living with HIV varies with the brain regions, being significant in the occipital pole and cerebellum but not in the frontal lobe (23). Accelerated aging of the occipital cortex (3.5 years) was associated with neurocognitive disorders (27). In adolescents who acquired HIV perinatally, age acceleration occurred in the blood cells (28) and is associated with brain development alterations (29). HIV-induced DNAme changes were partially restored with prolonged ART (30), which significantly reduces EA. Yet, in spite of the deceleration of HIV-induced age by ART (31), people living with HIV on ART still have a higher EA compared with uninfected individuals (32).

Even after decades of research, our understanding of HIV pathogenesis in relation to aging is incomplete, and further studies are needed to dissect the complex relationship between HIV and aging, two overlapping conditions with multiple clinical complications. Conducting clinical studies to understand the differences in the dynamics of HIV infection in the elderly versus younger populations poses multiple challenges because of the vast lifestyle differences between the elderly and the young population, such as diet, exercise, environment, comorbidities, and pharmacological treatments (33). Furthermore, assessing the impact of aging on the clinical and biological outcome of acute HIV infection might be difficult because of the inability to identify the exact time of infection and limited access to tissues. Animal models can provide an insight into the earlier stage of infection and its impact on age.

With the goal of examining the impact of age on HIV/SIV infection and its underlying mechanisms, we compared the dynamics of SIV infection in macaques (SIVmac) in aged versus young rhesus macaques (RMs). The aged RMs showed decreased survival and poor control of coagulation (D-dimer), systemic inflammation (C-reactive protein, CRP), and immune activation (soluble CD14, sCD14). Meanwhile, although the young RMs initially exerted better control of key aspects of pathogenesis compared with the old RMs, this advantage was offset with disease progression. The decline of control of disease progression in the young in the later stages of infection coincided with an EA increase in the PBMCs and with a stronger increase in EAA in the internal tissues, especially in the cerebellum and heart, in young versus aged RMs, suggesting that infection-related accelerated aging depends on host age.

## Results

**Figure 1:**
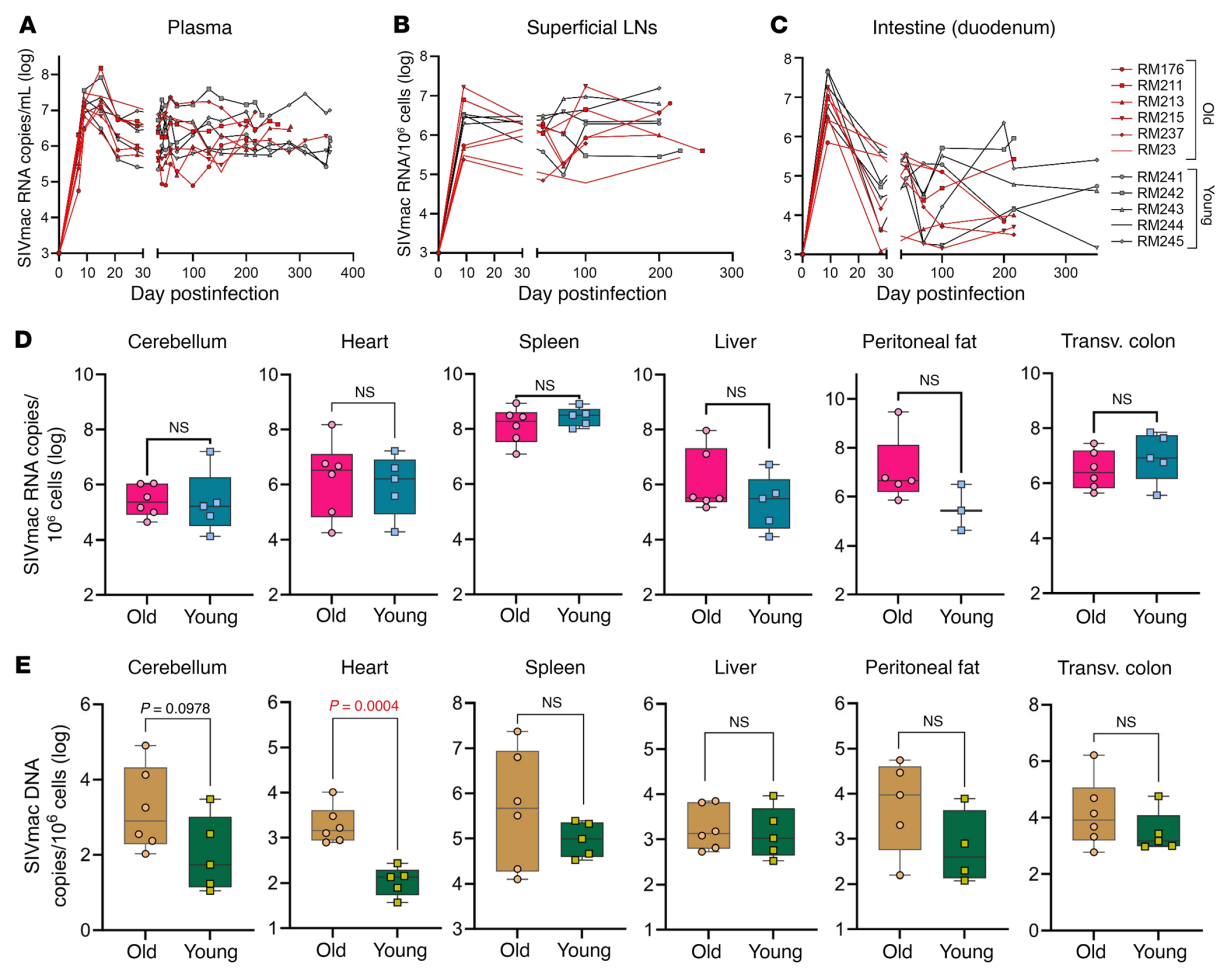
Viral RNA and DNA burdens. Dynamics of plasma viral loads (vRNA copies/mL) (**A**) and cell-associated viral RNA (CA-vRNA) (copies/10^6^ cells) in the superficial lymph nodes (SLNs) (**B**) and duodenum (**C**) in old (red lines) and young (black lines) rhesus macaques. CA-vRNA (**D**) and cell-associated vDNA (**E**) in necropsy tissues (cerebellum, heart, spleen, liver, peritoneal fat, transverse colon) from the advanced stage of infection. Significance was determined using a 2-tailed, unpaired *t* test.

**Figure 2:**
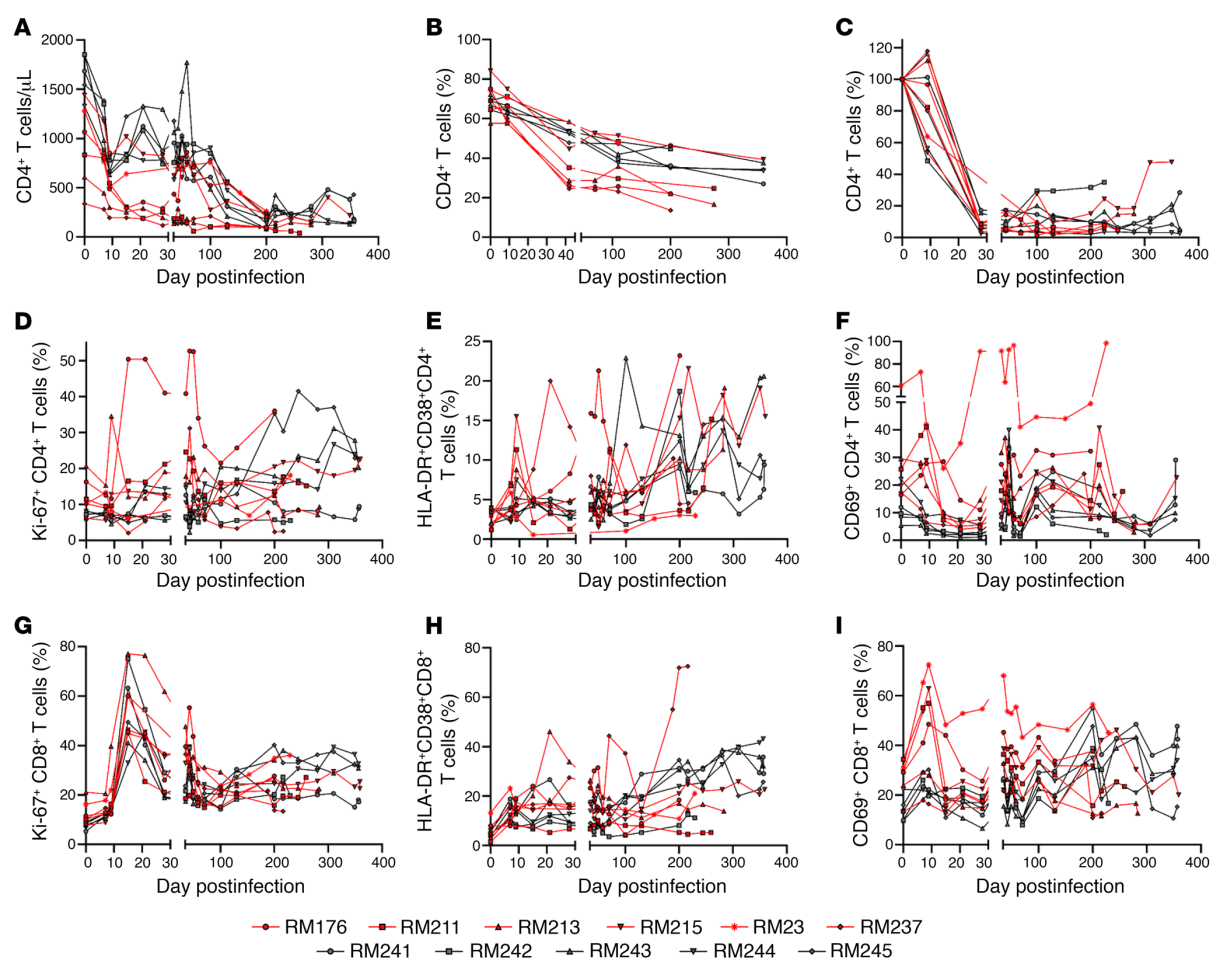
Longitudinal changes in CD4^+^ and CD8^+^ T cell profiles. Dynamics of CD4^+^ T cell counts in circulation (**A**), superficial lymph nodes (SLNs) (**B**), and gut (**C**). Changes in T lymphocyte proliferation assessed by measuring the expression of the fraction of circulating CD4^+^ T cells (**D**) and CD8^+^ T cells (**G**) expressing Ki-67. Dynamics of the expression of HLA-DR and CD38 on circulating CD4^+^ T cells (**E**) and CD8^+^ T cells (**H**). Dynamics of the expression of the early activation biomarker CD69 assessed on circulating CD4^+^ T cells (**F**) and CD8^+^ T cells (**I**). Old RMs are shown as red lines; the young ones as black lines.

**Figure 3:**
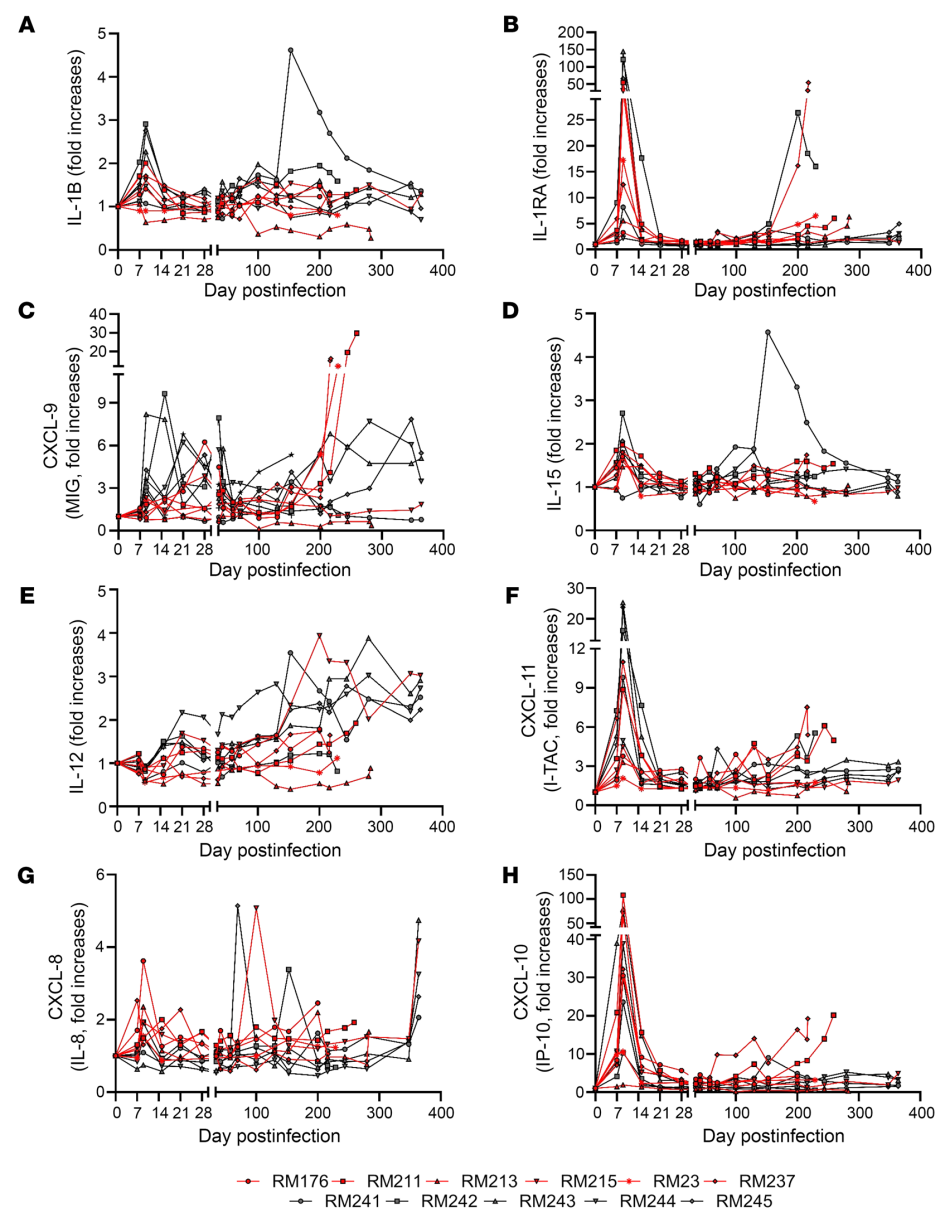
Changes in the plasma levels of inflammatory molecules in SIVmac239-infected old (red) and young (black) rhesus macaques: IL-1B (**A**), IL-1RA (**B**), CXCL-9 (MIG) (**C**), IL-15 (**D**), IL-12 (**E**), CXCL-11 (I-TAC) (**F**), CXCL-8 (IL-8) (**G**), and CXCL-10 (IP-10) (**H**).

**Figure 4:**
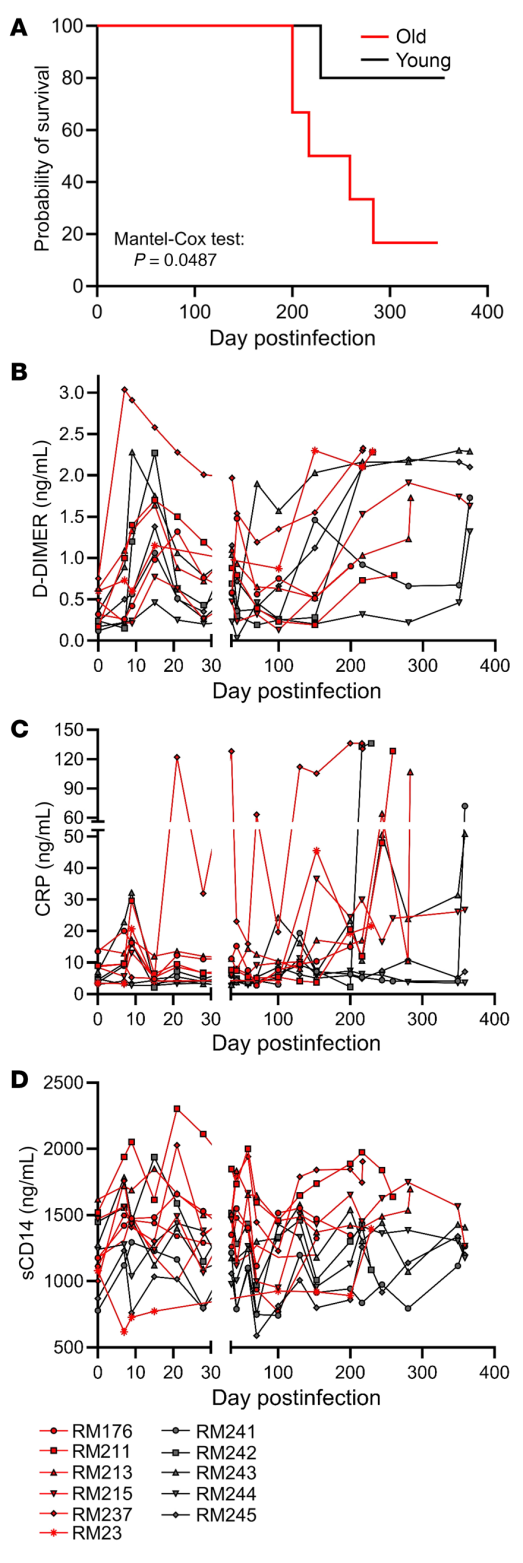
Probability of survival after SIV infection in old (red lines) versus young (black lines) rhesus macaques. (**A**). Changes in the levels of biomarkers that were reported to be predictive for HIV disease progression and death in SIVmac-infected old (red) and young (black) rhesus macaques: D-dimer (**B**); C-reactive protein (CRP) (**C**); and sCD14 (**D**).

To test the hypothesis that host age at the time of infection is critical to the course of SIV disease, the pace of host aging, and comorbidity risks, we longitudinally assessed its impact on the course of SIV infection. We included 6 aged RMs (15–22 years) to model elderly people living with HIV, and 5 young RMs (4–6 years) to model young adults (Table 1). RMs received 300 tissue culture ID_50_ SIVmac239 (34), and tissues critical for HIV/SIV pathogenesis were sampled at baseline and throughout infection until progression to AIDS (Table 2). We show the dynamics of each biomarker in Figures 1, 2, 3, 4 for individual values and in Supplemental Figures 1–7 for averages (supplemental material available online with this article; https://doi.org/10.1172/JCI189574DS1).

For the DNAme studies, we first examined a dataset of 40 PBMCs collected from 10 RMs throughout the follow-up: at the baseline and during acute, early chronic, and late chronic infection (Table 3). We also analyzed the DNAme profiles in 56 tissue samples from the gut (transverse colon), cardiometabolic tissues (liver, heart, and peritoneal fat), and immune tissues (spleen) obtained from 10 RMs during the late chronic infection (except for fat, which was only acquired from 6 RMs) (Table 3).

### Young SIV-infected RMs yield higher plasma viral loads than aged RMs due to a greater abundance of target cells.

Gradual depletion of CD4^+^ T cells and increasing plasma viral loads are major hallmarks of HIV/SIV disease progression (1, 35). CD4^+^ T cells decreased throughout infection both in the periphery (*P* < 2 × 10^–16^) and in the superficial lymph nodes (SLNs) (*P* = 1.5 × 10^–8^) (Supplemental Table 1). The young SIVmac-infected RMs exhibited slightly higher plasma viral loads than the aged ones (Figure 1A and Supplemental Figure 1A) and, to a lesser extent, in the SLNs and gut (Figure 1, B and C, and Supplemental Figure 1, B and C). The CD4^+^ T cells were significantly higher in the young compared with the old RMs in the periphery at the baseline (*P* = 0.0087) and during the early chronic infection (*P* = 0.043) (Supplemental Table 1).

The higher plasma viral loads observed in the young versus aged RMs were associated with higher baseline CD4^+^ T cell counts in the young RMs (Figure 2A and Supplemental Figure 2A). This trend was maintained throughout the early chronic infection due to an increased capacity to restore the CD4^+^ T cells by young RMs (Figure 2A and Supplemental Figure 2A). More robust plasma viral loads contributed to an accelerated CD4^+^ T cell loss in young RMs, and virtually complete depletion during chronic infection, similar in both groups. In the lymph nodes, both groups experienced CD4^+^ T cell depletions upon infection, slightly faster in aged RMs (Figure 2B and Supplemental Figure 2B). Finally, mucosal CD4^+^ T cell losses were similarly massive and swift in both groups, resulting in nearly complete depletion around 28 days after infection (dpi) (Figure 2C and Supplemental Figure 2C).

There was no clear association between age and the viral RNA/viral DNA content in most tissues (Figure 1, D and E), yet the aged RMs yielded a higher amount of SIV DNA in the heart (*P* = 0.0004) (Figure 1E) and a trend for higher virus replication in the cerebellum (*P* = 0.0978) (Figure 1E) compared with young RMs.

### Chronic T cell activation and proliferation increase in young RMs up to or above the levels observed in aged RMs.

Chronic activation drives CD4^+^ T cell loss and HIV/SIV disease progression (4). Meanwhile, aging-associated immune activation contributes to age-related diseases and increases the risk of uncontrolled inflammatory responses to infections (4). We monitored multiple CD4^+^ and CD8^+^ T cell activation markers: Ki-67, CD69, HLA-DR, and CD38 (Figure 2, D–I, and Supplemental Figure 2, D and I), and CD25 (Supplemental Figure 3, A–D). Aged RMs expressed higher baseline levels of activation markers: Ki-67 on CD4^+^ (*P* = 0.055) and CD8^+^ T cells (*P* = 0.03), CD69 on CD4^+^ (*P* = 0.03) and CD8^+^ T cells (*P* = 0.03), and CD25 on CD4^+^ and CD8^+^ T cells, yet without statistical significance (Figure 2, D, F, G, and I; Supplemental Figure 2, D, F, G, I; and Supplemental Figure 3, A–D). Conversely, CD38 and HLA-DR were similarly expressed by CD4^+^ and CD8^+^ T cells in the 2 groups (Figure 2, E and H, and Supplemental Figure 2, E and H).

Upon infection, Ki-67 and CD69 expression on CD4^+^ and CD8^+^ T cells and the CD8^+^ T cell fraction expressing CD38 and HLA-DR steeply increased in young RMs, reaching the levels observed in the aged RMs (Ki-67^+^, shown in Figure 2D and Supplemental Figure 2D, and HLA-DR^+^CD38^+^CD4^+^ T cells, shown in Figure 2E and Supplemental Figure 2E), or even exceeding them (Ki-67^+^, shown in Figure 2G and Supplemental Figure 2G; HLA-DR^+^CD38^+^, shown in Figure 2H and Supplemental Figure 2H; and CD69^+^CD8^+^ T cells, shown in Figure 2I and Supplemental Figure 2I; but not CD69^+^CD4^+^ T cells, for which increases were less discernible, as shown in Figure 2F and Supplemental Figure 2F). CD25 dynamics on CD4^+^ and CD8^+^ T cells were similar in young and old RMs (Supplemental Figure 3). Taken together, our data suggest that despite the initial advantage, young RMs catch up with or even surpass the old RMs with respect to CD4^+^ and CD8^+^ T cell activation.

The markers with higher levels in the old versus young RMs in early chronic infection, that is, Ki67^+^ on CD4^+^ (*P* = 0.078) and CD8^+^ (*P* = 0.038) T cells and CD69^+^CD8^+^ T cells (*P* = 0.0079) also showed a faster increase throughout the chronic infection stage in the young versus old RMs, that is, Ki67^+^ on CD4^+^ (*P* = 0.07) and CD8^+^ (*P* = 0.00056) T cells and CD69^+^CD8^+^ T cells (*P* = 8.4 × 10^–9^) (Supplemental Table 1). Overall, higher activation levels were observed in the old RMs for CD69^+^ on CD4^+^ (*P* = 0.0043) and CD8^+^ (*P* = 0.082) T cells.

### Greater increases of inflammatory cytokines occur in young SIV-infected RMs.

Inflammation occurs early in SIV/HIV infection and its persistence during chronic infection is associated with disease progression, even in people with ART-suppressed virus (4). We assessed the dynamics of inflammatory responses to SIVmac infection (Figure 3 and Supplemental Figure 4), and we report that both their acute and chronic increases tended to be more robust in young versus aged RMs (Figure 3, A, C, and F, and Supplemental Figure 4, A, C, and F), showing significant or near statistically significant results for IL-1B (*P* = 0.082), CXCL-9 (*P* = 0.068), and CXLC-11 (*P* = 0.027) in acute infection and significant results for IL-1B (*P* = 0.046) and CXCL-9 (*P* = 0.046) in early chronic infection (Supplemental Table 1). IL-8 did not follow this pattern, but yielded higher levels in the old RMs in acute and early chronic infection, trending toward significance (*P* = 0.064 and *P* = 0.065) (Figure 3G and Supplemental Figure 4G). However, some markers did not adhere to this pattern, for example, IL-1RA, IL-15, IL-12 or CXCL-10 (Figure 3, B, D, E, and H, and Supplemental Figure 4, A, B, D, E, and H).

### Accelerated progression to AIDS occurs in aged versus young SIV-infected RMs.

During the follow-up period, 4 of 6 old RMs progressed to AIDS (median survival: 269 days), as opposed to only 1 of 5 young RMs. We found a significantly lower probability of survival for aged RMs compared with the young ones (*P* = 0.0487) (Figure 4A). The main cause of death in the old RMs was interstitial pneumonia.

### Excessive coagulation and inflammation occur preferentially in young SIV-infected RMs, reaching levels similar to those observed in old RMs with advanced infection.

Systemic levels of coagulation, inflammation, and T cell immune activation predict SIV/HIV disease progression (4, 15, 16, 36, 37). We assessed multiple biomarkers: D-dimer, a biomarker of the hypercoagulation state (15) (Figure 4B and Supplemental Figure 5A); CRP, an acute-phase inflammatory marker (15) (Figure 4C and Supplemental Figure 5B); and sCD14, a biomarker of monocyte activation in response to microbial translocation (16) (Figure 4D and Supplemental Figure 5C). These markers also increase with age (38–40), so their baseline levels were higher in old versus young RMs, albeit they did not reach significance for D-dimer (*P* = 0.067) and CRP (*P* = 0.052) (Supplemental Table 1). All 3 markers significantly increased throughout infection: D-dimer (*P* = 4.3 × 10^–11^), CRP (*P* = 0.015), and sCD14 (*P* = 0.0032) (Supplemental Table 1). The timing and magnitude of D-dimer and CRP changes were different in young versus aged RMs, albeit the overall dynamics were similar, with acute peaks, partial control during early chronic infection, and gradual increases with disease progression (Figure 4, B–D). Early chronic sCD14 levels were significantly higher in old versus young RMs (*P* = 0.031). Although both groups largely managed to achieve a partial postacute control of D-dimer, CRP, and sCD14, the young RMs exhibited a more substantial control to nearly baseline levels.

### Monocyte subsets are similar in young versus old RMs with advanced SIV infection.

Monocytes are key innate immune effectors, monocyte/macrophage activation being a major determinant of inflammation/immune activation in HIV/SIV infection (41). Monocytes from people with HIV exhibit reduced counts and increased activation (41). Nonclassical monocytes contribute to an inflammatory phenotype in elderly people with HIV (41), and an increased proportion of intermediate monocytes is associated with cognitive impairments in people with HIV (42). We monitored the impact of infection on classical (CD14^++^CD16^–^); intermediate (CD14^+^CD16^+^); and nonclassical (CD14^dim^CD16^+^) monocytes (43). Classical monocytes decreased slowly during the late chronic infection stage in both aged and young RMs (Supplemental Figure 6A). Conversely, intermediate and nonclassical monocytes tended to rise with disease progression, more slowly in young RMs, eventually reaching similar levels (Supplemental Figure 6, B and C). Monocyte activation status, assessed by measuring sCD163 plasma levels (44), increased more robustly in young RMs, being partly controlled during chronic infection in both groups (Supplemental Figure 7). Meanwhile, the early chronic sCD14 levels were higher in old versus young RMs (Figure 4D and Supplemental Figure 5C).

Taken together, the young RMs initially had either advantageous baselines or greater capacity to control the initial impact of SIV infection on many (yet not all) pathogenesis biomarkers compared with old RMs. Thus, in the initial stages of SIV infection, the young RMs better preserved CD4^+^ T cells, had lower expression of CD4^+^ T cell (CD69) and CD8^+^ T cell (Ki67, CD69, CD38) activation biomarkers, and kept at bay hypercoagulation (D-dimer) and inflammation (CRP). Then, young RMs gradually lost these abilities during chronic infection, achieving an aged activated phenotype similar to that of old RMs by the end of the follow-up period.

### SIV-induced changes in DNAme.

HIV infection widely influences DNAme profiles across the human genome (45), yet the impact of SIV infection in the reference RM model remains unknown. We characterized the dynamics and tissue specificity of the host DNAme responses to SIV infection in the tissues involved in the pathogenesis of HIV-associated non-AIDS conditions in young and old RMs.

### Epigenome-wide association studies of the stages of infection in PBMCs.

**Figure 5:**
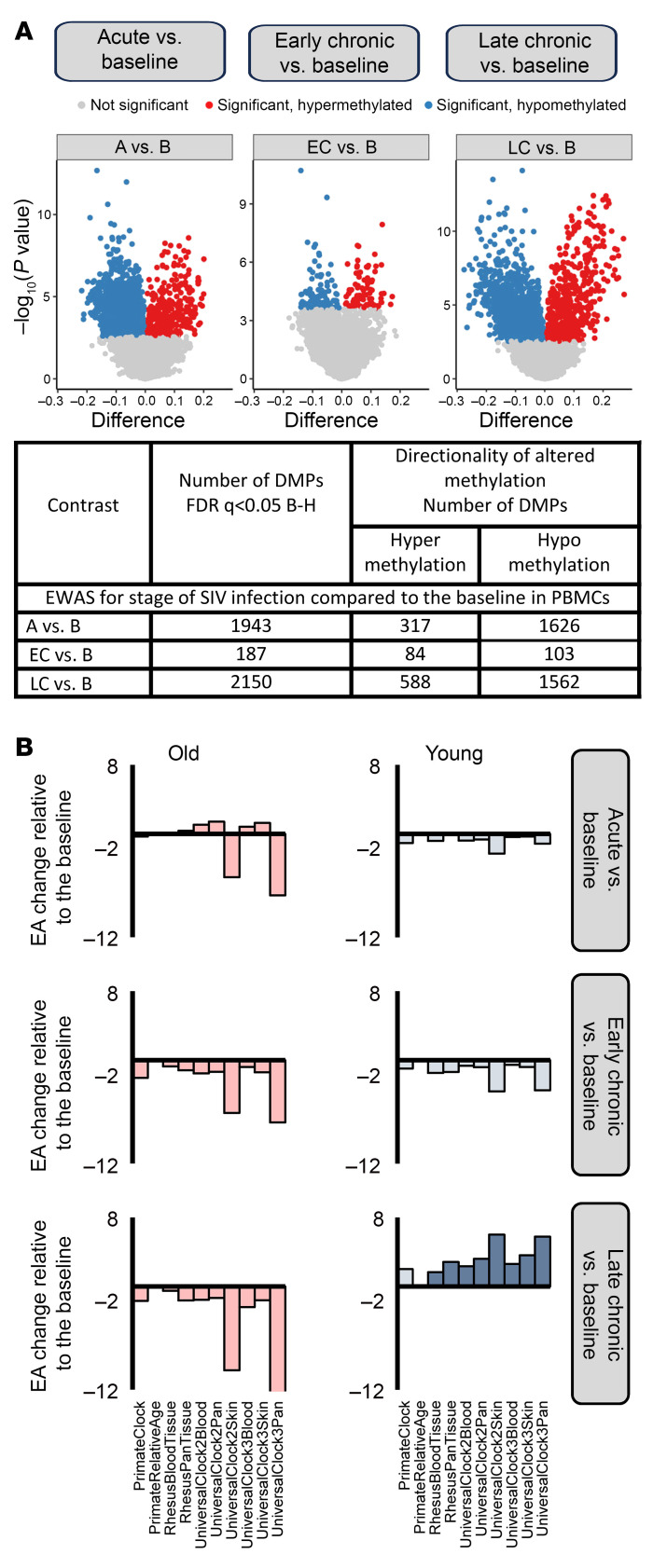
Longitudinal DNAme analysis of PBMCs. (**A**) Epigenome-wide association study of dpi in PBMCs based on the entire methylation array. The volcano plots display the −log_10_ (*P* values) and the directionality of association between CpG sites and infection stages (A, EC, and LC) compared with B: A versus B (left panel), EC versus B (center panel), and LC versus B (right panel). Each dot represents a specific DNAme site. Shown are significantly associated CpG sites (*q* < 0.05) with hypomethylation (blue), hypermethylation (red), and nonsignificant (gray). The horizontal axis represents the mean methylation change (i.e., the difference between group means), and the vertical axis represents −log_10_ (*P* values). (**B**) Changes in EA during each infection stage (A, EC, and LC) relative to B. Biological age analysis was performed based on subsets of clock CpGs. EA at the 3 infection time points was compared with B using mixed-effects linear regression modeling of longitudinal EA changes in PBMCs based on 10 epigenetic clocks. The results are shown separately for young (right) and old (left) RMs. Epigenetic age changes in young (blue) and old (red) RMs are shown. Saturated colors indicate statistically significant changes (*P* < 0.05); pale colors indicate nonsignificant changes (*P* > 0.05). A statistically significant increase in EA was observed only in young RMs. B–H, Benjamini–Hochberg correction; DMP, differentially methylated positions; dpi, days after infection; RMs, rhesus macaques; B, baseline; A, acute; EC, early chronic; LC, late chronic; EA, epigenetic age.

It is rarely possible to obtain samples prior to and during the acute HIV infection; therefore, we longitudinally assessed the effect of SIV infection on DNAme in RMs including these critical time points. We performed epigenome-wide association studies (EWAS) using the entire content of the methylation chip to analyze how SIVmac infection affects the global methylation profiles in the PBMCs in our aggregated group of young and aged RMs (Figure 5A and Supporting Data Values file).

Compared with baseline, deregulation of methylation of as many as 1,943 CpGs (associated with 890 genes) emerged during acute infection; the vast majority of CpG sites returned to the baseline during early chronic infection, and only 187 CpGs (associated with 126 genes) remained differentially methylated (Figure 5A). During late chronic infection, 2,150 CpGs (associated with 911 genes) underwent a new round of widespread aberrant methylation coincident with disease progression. There was a marked bias toward CpG hypomethylation: 83% during acute, 55% during early chronic, and 71% during late chronic SIV infection. Notably, these discernible shifts in DNAme occurred within 1 year of pathogenic infection, which is the timeframe in which the majority of SIVmac-infected RMs progress to AIDS (46). The most significant longitudinal changes in PBMC DNAme throughout infection were at the CpG sites associated with the *SPRED2* (hypomethylation) and *ZBTB7B*, also known as ThPOK (hypermethylation) genes.

We then used ingenuity pathway analysis (IPA) to longitudinally assess how methylation changes across biological pathways throughout SIV infection (47). The top canonical pathways associated with the acute infection included “aryl hydrocarbon receptor signaling,” the “role of BRCA1 in DNA damage response” pathway, and the “activin-inhibin signaling” pathway (–log_10_[*P*] > 1.3, Benjamini-Hochberg–corrected) (Supplemental Figure 8A). DNAme changes in acute SIV infection were enriched in a cluster of conditions related to liver tumorigenesis and congenital heart disease, whereas early chronic SIV infection was associated with dilated cardiomyopathy (Supplemental Figure 8B). DNAme shifts in acute and late chronic infection were enriched among various oncological categories and T cell malignancies (Supplemental Figure 9A); terms related to transcription regulation; lymphocyte homeostasis and proliferation; and differentiation, development, and homeostasis of T cells (Supplemental Figure 9B).

Given that SIV infection alters blood cell composition, mainly through the massive depletion of CD4^+^ T cells, we reran EWAS, accounting for the absolute CD4^+^ T cell counts in the blood. We observed considerably fewer significant EWAS hits (132 for acute vs. baseline, 47 for early chronic vs. baseline, and 76 for late chronic vs. baseline) (Supplemental Figure 10) in comparison to the results of EWAS without adjustment for CD4^+^ T cell population size, suggesting that CD4^+^ T cell depletion significantly contributes to the observed changes in global DNAme patterns.

### DNAme studies of biological age.

Epigenetic clocks predicting biological age allow assessment of the impact of various factors, including HIV, on aging (22, 23). To understand the impact of the host age on virus-driven aging, we determined the EA for each DNAme sample by using 10 epigenetic clocks previously developed based on the diverse tissue set and/or blood of primates and other mammals (48). EAA was determined as the difference between EA and chronological age.

### Links between EA, stage of infection, and biomarkers of pathogenesis in PBMCs.

We hypothesized that SIV infection of RMs, similar to HIV infection in humans, may affect EA. To test this hypothesis, we modeled the difference in the EA at each experimental time point compared with baseline in young and aged RMs by using a mixed-effects model that included the time point, sex, and age. A significant increase in the EA was observed during late chronic infection compared with baseline in young RMs but not in the old ones, based on 8 of 10 mammalian clocks (adjusted *P* < 0.05, Figure 5B). The concordance of predictions with 8 clocks implies that late-stage SIV disproportionately increases EA in the PBMCs of young RMs.

Multiple factors can affect biological aging, including infection stage, host age and sex, and ongoing pathogenic processes. We used variance analysis to evaluate the associations between EA, predicted based on the Universal Blood Clock 3 developed for blood and the key markers of SIV/HIV pathogenesis, including disease progression (CD4^+^ T cell counts, plasma viral loads, immune activation, and inflammation), microbial translocation, and hypercoagulation. We considered EA as a dependent variable and modeled it as a function of infection stage, age, sex, and individual. We tested whether including each biomarker in the model significantly improved the EA predictions.

**Figure 6:**
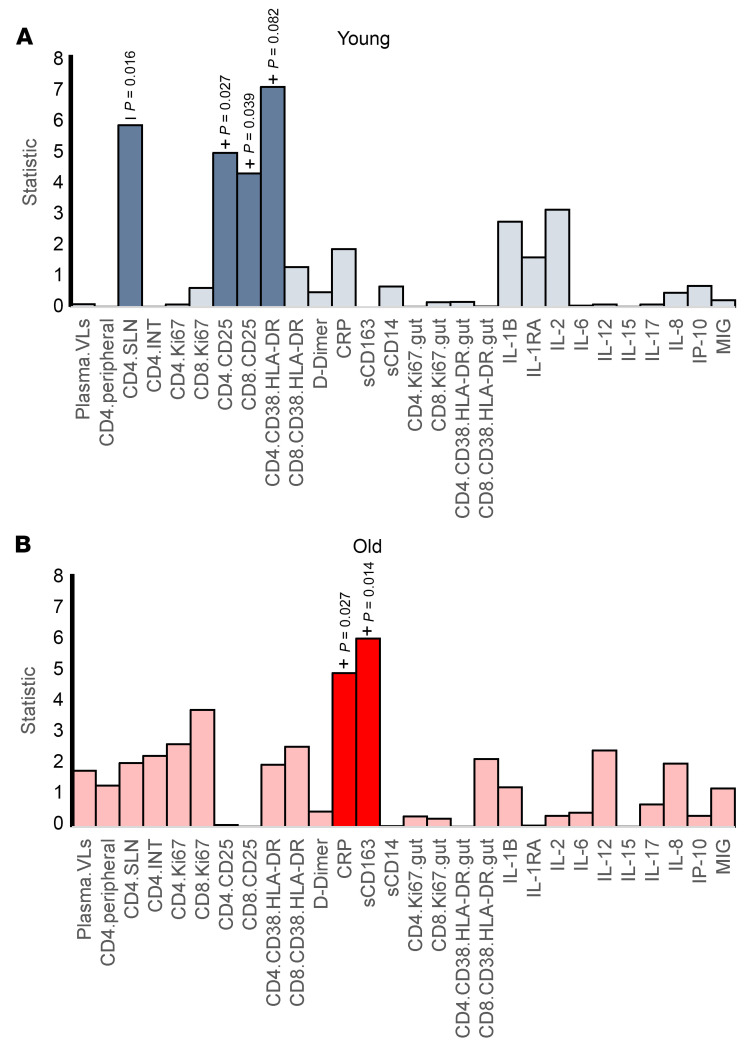
Biomarkers associated with epigenetic age in PBMCs across infection stages. *P* values above bars were obtained from a 2-sided ANOVA analysis comparing a full mixed-effects model, which included biomarker levels (measured at the time point closest to the clock measurements) as independent variables to a null model. The full model accounted for time point (B, A, EC, and LC), age, sex, and given biomarker; the null model excluded biomarker levels. For significantly associated markers, *P* values are shown on the plot, along with an indication of the directionality of the association (positive or negative). The results for young (**A**, blue) and old (**B**, red) RMs are shown. Saturated colors indicate statistically significant changes (*P* < 0.05); pale colors indicate nonsignificant changes (*P* > 0.05). Biomarker levels were measured during infection stage corresponding to clock measurements: 0 dpi, B; 9–21 dpi, A stage; 42–49 dpi, EC stage (except the immune activation markers in the gut, measured at 70 dpi); and more than 130 dpi, LC stage. B, baseline; A, acute; EC, early chronic; LC, late chronic; dpi, days after infection; RMs, rhesus macaques.

In young RMs, the EA of the circulating PBMCs was negatively associated with the abundance of CD4^+^ T cells in SLNs (*P* = 0.016) and positively associated with systemic T cell activation, that is, CD25^+^CD4^+^ T cells (*P* = 0.027), HLA-DR^+^CD38^+^CD4^+^ T cells (*P* = 0.0082), and CD8^+^CD25^+^CD8^+^ T cells (*P* = 0.039) (Figure 6A). In old RMs, EA was positively associated with the plasma levels of CRP (*P* = 0.027) and sCD163 (*P* = 0.014) (Figure 6B).

### Tissue-specific differences in EAA associated with SIV infection.

**Figure 7:**
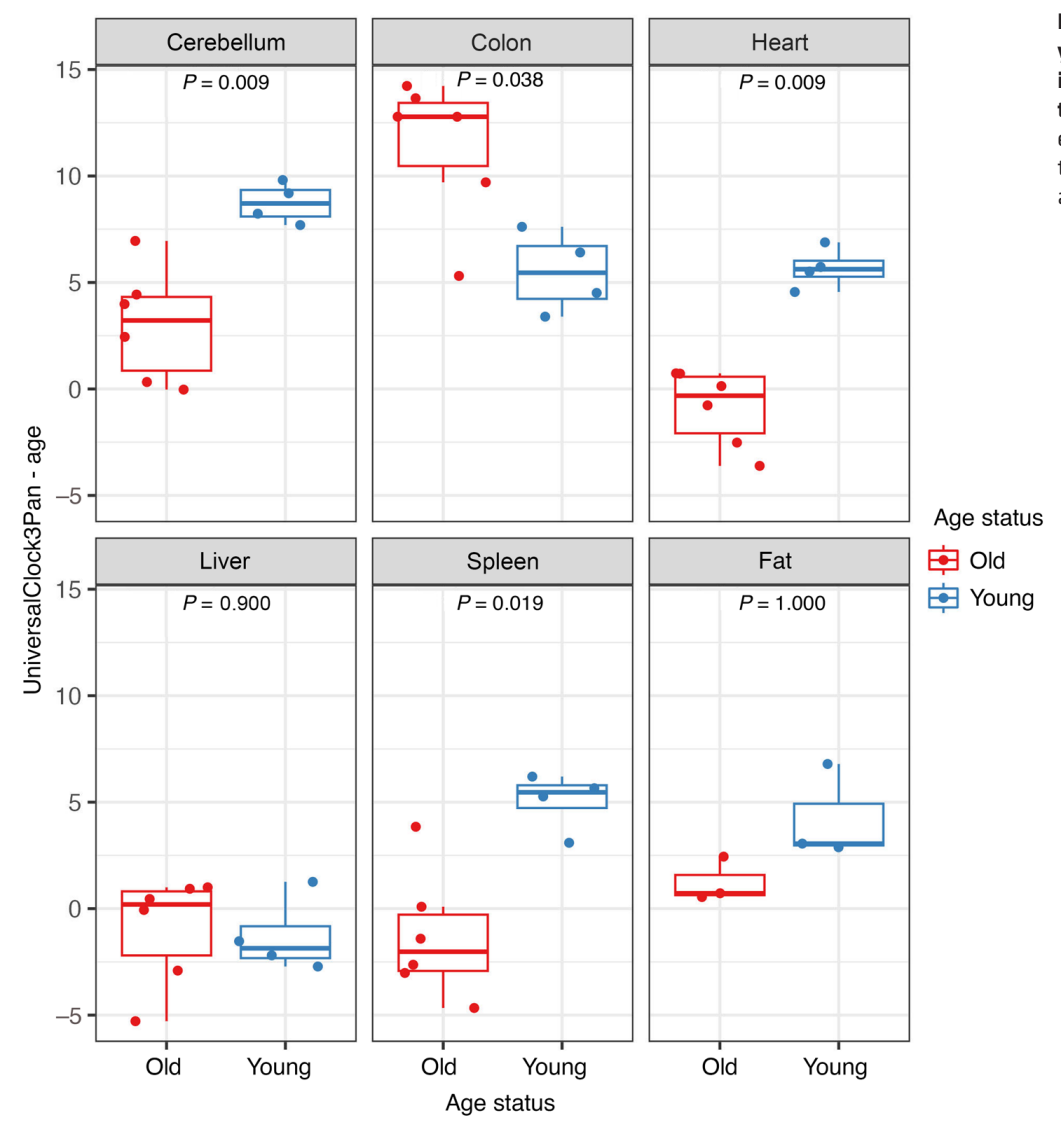
Epigenetic age acceleration in young (blue) and old (red) rhesus macaques in late chronic SIVmac infection in 6 tissue types based on Universal Clock 3. The differences in epigenetic age acceleration between the young and old rhesus macaques were assessed using a 2-sided Wilcoxon test.

To assess the impact of SIV infection on tissue aging, we used the Wilcoxon test and compared EAA between young and old RMs in 6 solid tissues during advanced-stage infection (Figure 7). EAA estimates based on the third universal pan-mammalian epigenetic clock (Universal Clock 3), the most robust multitissue pan-mammalian clock, indicated greater EAA in the young RMs versus aged RMs, particularly in the cerebellum, heart, and spleen, whereas the colon exhibited greater EAA in the aged versus young RMs. When applying 9 other epigenetic clocks, EAA increased more prominently in the young RMs in the cerebellum (per all 10 clocks), heart (per 9 of 10 clocks), spleen (per 5 of 10 clocks), liver (per 2 of 10 clocks), and colon (per the Primate Relative Clock) (Wilcoxon, *P* < 0.05) (Supplemental Figure 11). We did not observe EAA in the adipose tissue of RMs, but a smaller number of samples of this tissue was analyzed.

**Figure 8:**
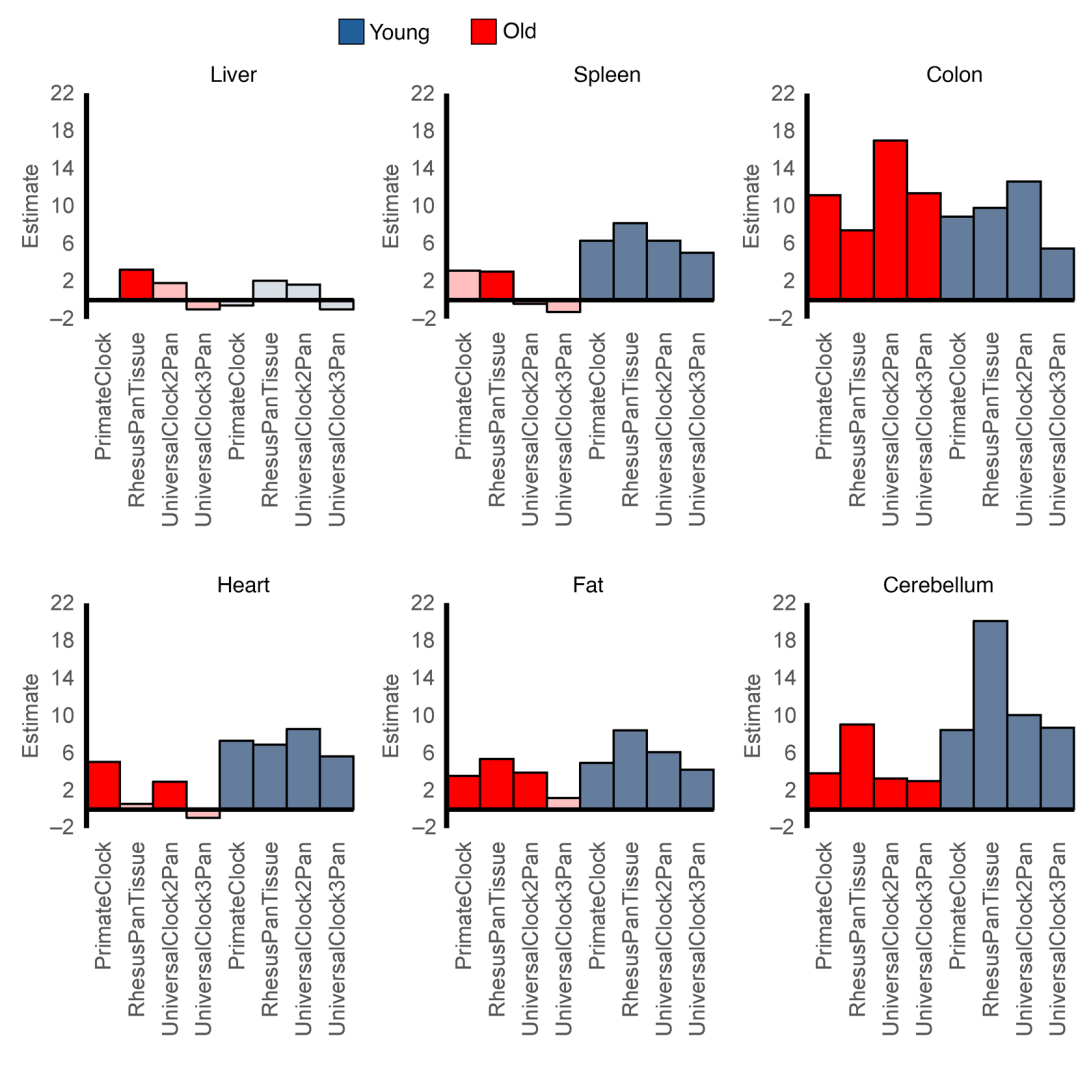
Effects of age (young or old) on epigenetic age acceleration in tissues collected at necropsy from young and old rhesus macaques during late chronic SIV infection. The results for young (blue) and old (red) rhesus macaques are shown. Saturated colors indicate statistically significant differences (*P* < 0.05); pale colors indicate nonsignificant differences (*P* ≥ 0.05). The effects were assessed using linear regression analysis, where epigenetic age acceleration was modeled as a function of age status (young or old). The clock measurements were obtained from tissues collected during necropsies in the late chronic infection stage (225–360 dpi for old and 366–380 dpi for young). Dpi, days after infection.

We next assessed the effect of age status on EAA in solid tissues with a subset of 4 epigenetic clocks most representative of internal tissues (Figure 8). The clocks concordantly pointed to a positive effect of age status on EAA in the colon, cerebellum, spleen, fat, and heart in the young RMs and in the colon, cerebellum, and fat in the aged RMs. These findings suggest that susceptibility to virus-driven EAA is tissue-specific and that the host’s age is a risk factor differentially influencing different tissues. Although the colon has a high cell turnover, the heart and cerebellum comprise extremely long-lived cells, which in the long-term may be particularly vulnerable to HIV-associated non-AIDS comorbidities.

### Correlation of biomarkers of SIV pathogenesis and EAA in tissues during the late chronic infection stage.

Given that HIV pathogenesis biomarkers are associated with EAA in the circulation and the gut (49), we calculated Spearman’s rank correlation between EAA measured using Universal Clock 3 in the solid tissues and circulating biomarkers of SIV pathogenesis, including CD4^+^ T cells, viral loads, T cell activation and proliferation status, inflammation, and hypercoagulation. We also analyzed the relationship between the estimated SIV DNA and RNA and EAA for each tissue, but did not find any significant associations.

**Figure 9:**
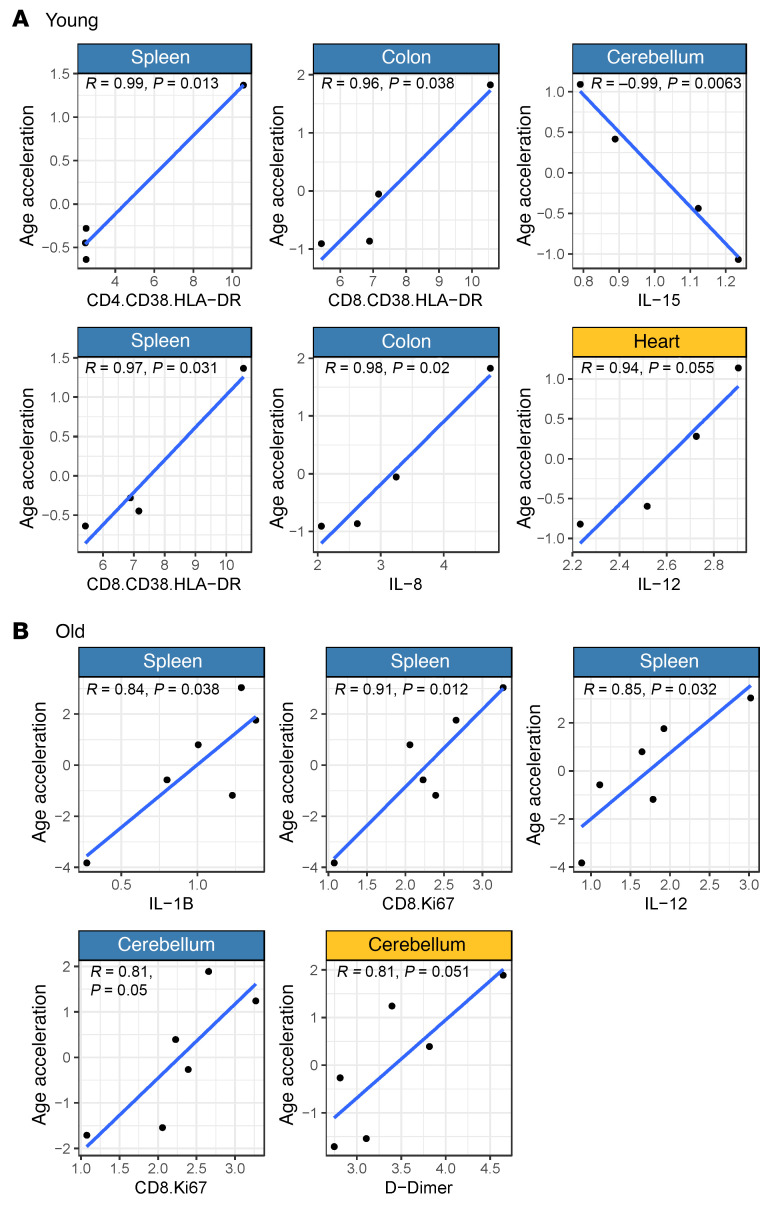
Association between tissue epigenetic age and peripheral pathogenesis markers. Correlation between biomarkers of immune activation, inflammation, coagulation, and cell-associated viral DNA and epigenetic age acceleration in tissues collected from young (**A**) and old (**B**) rhesus macaques during late SIV infection. Tissues for clock estimates were obtained during necropsy. Measurements of biomarker levels were obtained either from the necropsy or the nearest available time point (between 200 dpi and necropsy). Pearson’s correlation was used to assess these relationships. Statistically significant correlations (*P* < 0.05) are shown in blue; nonsignificant correlations (*P* > 0.05) are shown in yellow. Dpi, days after infection.

High levels of circulating markers of immune activation were positively correlated with EAA in tissues (Figure 9). In the young RMs, HLA-DR^+^CD38^+^CD4^+^ T cells correlated with EAA in the spleen (*P* = 0.013), and HLA-DR^+^CD38^+^CD8^+^ T cells correlated with EAA in the spleen (*P* = 0.031) and colon (*P* = 0.038) (Figure 9A). Conversely, in aged RMs, Ki-67^+^CD8^+^ T cells positively correlated with EAA in the spleen (*P* = 0.012) and displayed a positive trend with EAA in the cerebellum (*P* = 0.05). Taken together, these results imply a relationship between EAA and CD4^+^ and CD8^+^ T cell activation in young RMs and CD8^+^ T cell expansion in aged RMs (Figure 9B).

We also found that several inflammatory markers correlated with the rate of EAA. In old RMs, EAA in the spleen was directly related to the plasma levels of IL-1B, a key mediator of inflammatory response (*P* = 0.038), and IL-12, a biomarker of cardiovascular disease (50) and promoter of spleen hematopoiesis (*P* = 0.032) (51). Also, D-dimer exhibited a positive correlation trend with EAA in the cerebellum in the old RMs (*P* = 0.051).

In young RMs, colon EAA correlated with plasma levels of IL-8 (a biomarker of chronic inflammation acting as a neutrophil chemoattractant that stimulates neutrophil phagocytic activity; ref. 52; *P* = 0.02), whereas EAA in the heart exhibited a positive correlation trend with plasma IL-12 levels (*P* = 0.055). IL-15 exhibited the strongest correlation among the plasma biomarkers, and was the only biomarker negatively correlated with tissue EAA. Taken together, our results indicate the role of systemic inflammation (increased IL-1 and IL-12 levels) in the aging process and potential splenic dysfunction in aged RMs. In young RMs, aging of tissues with low cell turnover (i.e., heart and cerebellum) was related to IL-15 and IL-12 deregulation.

## Discussion

Natural aging and HIV/SIV infection–triggered accelerated aging have several common features: progressive loss of CD4^+^ T cells, chronically increased T cell immune activation and inflammation, a hypercoagulable state, and increases in microbial translocation. Given that host ages are critically shaping infection outcomes in general (53), we anticipated that SIV-accelerated aging would have a major impact on infection outcome.

During HIV infection, CD4^+^ T cell counts decrease due to virus- or immune activation–mediated CD4^+^ T cell destruction and impaired lymphopoiesis, which occur even in virologically suppressed people living with HIV (54). CD4^+^ T cell counts declined with SIV infection in both young and aged RMs. At the baseline, the young RMs naturally had a greater abundance of CD4^+^ T cells, supporting the notion that aging affects T cell homeostasis. The low CD4^+^ T cell counts in older individuals have multiple causes: with age, the thymic output becomes mainly constrained to the peripheral proliferation of naive and memory T cells (54). Furthermore, naive T cells are gradually replaced by memory cells (54). These differences in the CD4^+^ T cells and the host capacity to replenish them not only drove the dynamics and magnitude of their depletion during SIV infection, but may have also affected viral replication, which was more robust in younger RMs in the initial stages of infection.

In the old animals, inability to restore CD4^+^ T cells was associated with a more profound immune suppression, opportunistic infections, and increased SIV-related mortality. The levels of predictive biomarkers of HIV-related mortality (D-dimer, CRP, and sCD14) were initially closer to baseline in the young RMs. However, in late chronic infection, D-dimer and CRP increased sharply in the young RMs infected with SIV, reaching levels close to those observed in old RMs. Although our follow-up period was too short and we euthanized the young RMs after the old ones died of AIDS, this may indicate an increased incidence in young people of early-onset vascular HIV-associated non-AIDS conditions.

In the young RMs, similar dynamics were observed for markers of T cell proliferation and immune activation, which were initially controlled, yet ended by approaching or exceeding the levels observed in aged individuals with disease progression. Thus, young RMs appear to have an intrinsic advantage during early infection, when they can partially control key mechanisms driving SIV disease progression. However, not all markers followed this pattern in the young RMs. IL-12 and IL-1B showed more robust increases in young versus old RMs, suggesting that different types of inflammatory responses may protect the young animals against early disease progression.

In the young RMs, CD4^+^ T cell depletion and CD4^+^ and CD8^+^ lymphocyte activation were associated with EA in the PBMCs. Also, tissue EAA (spleen and colon) correlated during late chronic infection with the biomarkers of circulating lymphocyte activation. In advanced infection, plasma cytokines positively correlated with tissue EAA in young RMs: IL-8 (colon) and IL-12 (heart). Conversely, IL-15 showed an anticorrelated pattern with the EAA in the cerebellum. This observation aligns with reports of age-related IL-15 downregulation (55), positive associations with ultra-longevity, and increases of CD8^+^ memory T cell longevity by inducing telomerase activity (55). The observed reduction of IL-15 with EAA may affect the control of SIV/HIV infection, as IL-15 enhances memory T cell survival, particularly CD8^+^ T cells (56); stimulates HIV/SIV-specific cytotoxic T lymphocyte expansion (57); and promotes the expansion and differentiation of natural killer cells and macrophage maturation (55).

With regard to the molecular mechanisms of accelerated aging, we showed that EA significantly increased in PBMCs during late chronic infection in young but not in aged RMs. An increase in EAA in the young versus aged RMs was observed across most of the tissues studied, with the most consistent support from different clocks for the cerebellum and heart, followed by the spleen. The RM model of AIDS reproduced the accelerated aging previously reported to occur in people living with HIV (29, 58), particularly the brain (cerebellum) (23) and the spleen (59). In the old adults, the colon was the only tissue yielding higher EAA compared with the young RMs. This may be related to a potential age-related decline in repair capacities due to the damage acquired through prolonged exposure to inflammation, loss of barrier function, and/or shifts in microbiome diversity and composition (60, 61). We report that infection-driven age acceleration is not only tissue-specific but also depends on the age of the host, with young adults having an increased risk of accelerated aging, particularly in the blood, cerebellum, and heart.

The biological aging in PBMCs, which have a relatively rapid turnover and possibly very dynamic proportions, may reflect aging of the blood cells or their stem cells, shifts in cellular composition (e.g., altered CD4^+^ and CD8^+^ T cell subsets), and/or changes in cellular phenotypes (e.g., T cell dysfunction and exhaustion). By contrast, the structure and function of the brain and heart depend mainly on cells with extremely low renewal rates and long lifespans, which are practically lifelong (62, 63). Therefore, the observed aging-related profiles in these tissues rather reflect epigenetic changes in the cells themselves rather than shifts in cellular composition.

In PBMCs, widespread changes in bulk DNAme profiles occurred early during the acute infection stage. The DNAme profiles partly overlapped during the acute and terminal infection stages, whereas in the early chronic infection stage, they virtually returned to preinfection baseline. The most significant methylation changes were associated with the *SPRED2* gene (hypomethylated), which negatively regulates innate immune responses by inhibiting the ERK–MAPK pathway and IFN-γ production by T cells (64, 65) and the *ZBTB7B* gene (hypermethylated), which is a key differentiation factor during positive selection that mediates CD4^+^ T cell commitment and prevents CD8^+^ T cell commitment (66, 67). Although we could not fully adjust EWAS for cellular heterogeneity because of unavailability of a reference set suitable for our model, we observed that changes in circulating CD4^+^ T cell abundance had a strong effect on the bulk DNAme profiles.

SIV infection was associated with differential DNAme profiles in or near several genes involved in the regulation of HIV replication and silencing mechanisms of HIV latency, including *DDX5* (68, 69) and *PDCD1* (70), as well as genes related to T cell commitment, including *ZBTB7B* fostering CD4^+^ T cell commitment and *LEF1*, which represses CD4^+^ T cell lineage genes, promoting CD8^+^ T cell identity (71). The differential DNAme also comprised key gene expression modulators, such as *TNRC6B*, a component of the miRISC, and *ZEB2*, a key transcription factor in embryogenesis, wound healing, and hematopoietic differentiation (72) and epigenetic modulator of the transcription of atherosclerosis-associated genes (73).

In PBMCs, acute SIV infection was associated with differential DNAme of genes in the signaling pathway of the aryl hydrocarbon receptor that is activated through the oncometabolite kynurenine, associated with increased tryptophan catabolism by indoleamine 2,3-dioxygenase and modulation of immune responses and inflammation (74, 75). It controls the expression of several immunosuppressive signaling molecules involved in the immunological barrier function of different organs and mediates host-microbiome communication (76, 77). The aryl hydrocarbon receptor influences T cell differentiation and function, promoting HIV infection and reactivation (78) while blocking HIV-1 replication in macrophages (79).

The main pathways for which gene methylation status changed in response to HIV-1 infection were related to common comorbidities: cancer, cardiovascular diseases, fibrosis, and gastrointestinal diseases. For example, cardiomyopathy was among the IPA functions connected to early chronic infection in PBMCs, and it is known that HIV causes a twofold increased risk of cardiovascular disease (80); raises the risk of heart failure (81), interstitial cardiac fibrosis, and sudden cardiac death (82); and increases the occurrence of and death from arrhythmia (83). On the cellular level, DNAme changes in the acute and late chronic infection periods were enriched in the genes related to immune cell proliferation, differentiation, and homeostasis, including T cells.

In summary, we demonstrated that (a) host age is an important factor shaping survival, physiological responses to SIV infection in RMs, and the EA in various tissues involved in HIV pathogenesis, including PBMCs, cerebellum, heart, and spleen, which are also affected by accelerated aging in people living with HIV, and (b) SIV infection exerts massive changes on the DNAme level, including genes essential for processes contributing to HIV/AIDS disease and HIV-associated non-AIDS conditions. As such, the RM model can be used for detailed mapping of gene changes to identify gero-protective therapeutics to improve the quality of life for people living with HIV.

SIVmac infection and disease progression in RMs are accompanied by a wide variety of epigenetic changes. We provide direct evidence that pathogenic SIV infection drives age acceleration in RMs, similar to HIV-1 infection in people, thus offering a mechanistic explanation for the development of multiple comorbidities in people living with HIV and providing a suitable translational model for development and preclinical testing of antiaging strategies against virus-induced age acceleration in HIV/AIDS.

## Methods

### Sex as a biological variable.

RMs of both sexes were included in this study. The proportion of females in the group of old RMs was 5 of 6 and in the group of young RMs was 3 of 5. Sex cannot be studied as a biological variable in such small groups of monkeys.

### Ethics statement.

Indian RMs involved in this study were obtained from Alpha Genesis and were housed at the Plum Borough Research Center of the University of Pittsburgh. Study animals were socially housed (paired) indoors in stainless steel cages, had a 12-hour light/12-hour dark cycle, and were fed twice daily; water was provided ad libitum. Environmental enrichment strategies were employed, including paired housing, toys to manipulate, and entertainment videos in the animal rooms. RMs were observed twice daily. Signs of disease or discomfort were reported to the veterinary staff for evaluation. For sampling, animals were anesthetized with 10 mg/kg ketamine HCl (Park-Davis) or 0.7 mg/kg tiletamine HCl and zolazepam (Telazol, Fort Dodge Animal Health) injected intramuscularly. Upon study completion, RMs were euthanized by intravenous administration of barbiturates.

### Animals, infections, and sampling.

Eleven adult Indian RMs (*Macaca mulatta*), 6 aged RMs (average age: 18.61 years) and 5 young RMs (average age: 5.08 years) (Table 1) were infected with 300 tissue culture ID_50_ SIVmac239, as described previously (34), and followed until they progressed to AIDS. All but 2 aged RMs were monitored throughout acute and chronic SIV infection for 1 year, or until they progressed to AIDS, when they were euthanized. The remaining 2 RMs were followed until 142 dpi, and then transferred to other studies. The study design with key sampling points is shown in Supplemental Figure 13.

### Samples.

Plasma was separated from whole blood by centrifugation (1,500*g*, 20 minutes), and PBMCs were isolated using a Ficoll density gradient centrifugation. PBMCs were pelleted, and 2 × 10^6^ cells were dry-frozen.

Serial biopsies of the colon and SLNs were collected as described previously (84, 85). Intestinal biopsies were washed with EDTA for 20 minutes at 37°C, and then subjected to collagenase digestion at 37°C with agitation. Cell suspension was filtered through a 70 μm filter, and then layered in a tube containing 2 mL of 35% Percoll solution on top of 2 mL of 60% Percoll solution. After a centrifugation at 1,500*g* for 20 minutes, lamina propria lymphocytes were retrieved at the interphase between the 2 Percoll solutions. SLN lymphocytes were separated by mincing the tissue, followed by pushing through a 70 μm nylon mesh screen, and then filtered through 70 μm nylon mesh bags and washed with RPMI medium (Cellgro) containing 5% heat-inactivated newborn calf serum, 0.01% penicillin-streptomycin, 0.01% L-glutamine, and 0.01% HEPES buffer. Freshly isolated cells were then used for flow cytometry.

### Plasma viral load quantification.

Quantification was performed on all the blood samples collected at all time points using a reverse-transcription real-time PCR (qRT-PCR), as described previously (34, 86, 87), utilizing an ABI 7900 HT and SDS v2.4.1 software (Applied Biosystems) (34).

### Cell-associated viral RNA and DNA.

PBMCs and tissues collected at necropsy were quantified for viral RNA and proviral DNA by qRT-PCR. Total RNA was extracted from cell pellets using a TRIzol-based protocol. Briefly, 400 μL Tri-reagent (Molecular Research Center, Inc.) was added, followed by vortexing until pellets were dissolved. Next, 100 μL 1-bromo-3-chloropropane (Molecular Research Center, Inc.) was added to each tube, vortexed for 15 seconds, and centrifuged at 14,000*g* for 15 minutes at 4°C. The upper aqueous RNA phase was removed, added to 12 μL of 20 mg/mL glycogen, and mixed by pipetting, followed by the addition of 500 μL isopropanol, mixing, and centrifugation at 21,000*g* for 10 minutes at room temperature. Isopropanol was removed, and RNA pellets were washed with 70% ethanol and kept overnight at –20°C to ensure complete leaching of salts from the pellets. The following day, the ethanol was removed by centrifugation at 14,000*g* for 5 minutes, and pellets were resuspended in 5 mM Tris. The DNA phase was extracted by adding 500 μL of Back extraction buffer (4 M guanidine thiocyanate, 50 mM sodium citrate NaCL, 1 M Tris). Samples were vortexed for 15 seconds and centrifuged at 14,000*g* for 15 minutes at 4°C. The upper phase was collected, and 12 μL of glycogen (20 mg/mL) and 400 μL isopropanol were added to each sample. Samples were mixing and spun at 14,000*g* for 10 minutes at room temperature. After removal of isopropanol and an overnight wash with 70% ethanol at 4°C, samples were centrifuged at 14,000*g* for 5 minutes and the DNA pellets were resuspended in 5 mM Tris.

For all the viral quantification assays, extracted RNA samples were reverse-transcribed, and both the cDNA and the viral DNA were quantified by qPCR using the forward primer 5′-GTCTGCGTCATCTGGTGCATT-3′; reverse primer 5′-CACTAGGTGTCTCTGCACTATCTGTTTTGC-3′; and probe 5′6-FAM/CTTCCTCAG/ZEN/TGTGTTTCACTTTCTCTTCTGCG/3I′ABkFQ. To determine the cell count per sample, CCR5 was quantified, and the final number of viral RNA and proviral DNA copies was divided by the number of cells from the sample to determine the number of viral RNA or DNA copies per million cells. All reactions were performed in duplicate on LightCycler 480 (Roche Diagnostics) at 95°C for 5 minutes, and then 50 cycles of 95°C for 15 seconds, 60°C for 1 minutes, and a hold step at 37°C.

### Genomic DNA from longitudinally collected PBMCs.

Genomic DNA was extracted by cell lysing PBMC pellet with RLT buffer and shearing the lysate on the shredder columns, and then performing the AllPrep DNA/RNA protocol (QIAGEN). Solid tissues (SLNs, mesenteric lymph nodes, jejunum, ileum, transverse colon, cerebellum, liver, heart, peritoneal fat, and spleen) were collected during necropsies. Genomic DNA was isolated from the solid tissues using the Tri Reagent (Sigma-Aldrich) protocol with homogenization using zirconia silica ceramic beads in the Geno/Grinder (Spex), with further purification using Genomic DNA Clean & Concentrator (Zymo).

### Flow cytometry.

Whole blood was stained longitudinally throughout the follow-up period to assess the impact of SIV infection on the dynamics of major immune cell populations and T cell immune activation. TruCount (BD Biosciences) was used to enumerate the absolute counts of circulating CD4^+^ and CD8^+^ T cells (87). Blood was stained with fluorescently labeled antibodies (from BD Biosciences, except where noted): CD3 (V450), CD4 (L200), CD8 (RPA-T8), CD25 (2A3), CD69 (FN50), CD38 (HB7), HLA-DR (L243), CD14 (M5E2, BioLegend), and CD16 (3G8). In addition, Ki-67 staining (B56, BD Biosciences) of CD4^+^ and CD8^+^ T cells was performed, with postfixing and permeabilizing of cells after the surface staining. Flow cytometry acquisitions were done on an LSR II flow cytometer (BD Biosciences, with FACSDiva v8.01 and FlowJo v10.7.1). All antibodies were used according to the manufacturers’ recommendations, and validation for use of these antibodies in RMs was ascertained by the NIH (as indicated in www.nhpreagents.org/ReactivityDatabase).

### ELISA and Luminex assays.

Multiple soluble markers were quantified in the plasma of RMs by multiple immunoassays: sCD14 (Quantikine Human sCD14 Immunoassay, R&D Systems); sCD163 (Macro163, IQ Products); and CRP (Monkey CRP ELISA kit, Life Diagnostics). D-Dimer was measured with an immunoturbidimetric assay (Liatest D-DI, Diagnostica Stago) using a STAR automated coagulation analyzer (Diagnostica Stago).

Expression of cytokines and chemokines was quantified by measuring their plasma levels using a magnetic bead-based assay, the Cytokine 29-plex Monkey Panel (Thermo Fisher Scientific). Plates were read on a MAGPIX. All ELISA and Luminex assays were performed according to the manufacturer’s instructions.

### DNAme.

DNAme was assessed on DNA extracted from 2 sample sets: (a) 6 solid tissues (cerebellum, trans colon, liver, heart, peritoneal fat, and spleen) sampled at necropsy (197–380 dpi); and (b) a longitudinal set of 40 PBMC samples collected from 5 aged and 5 young RMs at the baseline and 3 time points (detailed in the experimental design in Supplemental Figure 13). DNAme profiles were generated using a custom Infinium methylation array (HorvathMammalMethylChip40) representing 37,492 CpG highly conserved sites in the mammals, with the NCBI’s Gene Expression Omnibus (GEO) accession number GPL28271 for microarray design (88). The CpG probe annotations specific to the RM genome were acquired (https://www.github.com/shorvath/MammalianMethylationConsortium/). We used the entire set of 35,053 probes on the array that map to the RM genome, including CpGs located in and within proximity to 5,773 RM genes. DNAme profiles from tissue and PBMC DNA were generated by the UCLA Neuroscience Genomics Core. Bioinformatic analysis was conducted by the Epigenetic Clock Development Foundation and deposited in the NCBI’s GEO under accession numbers GSE226899 (tissues) and GSE226858 (PBMCs). Beta values and detection *P* values were determined using SeSaMe (89).

All DNAme tissue and PBMC samples were clustered based on their pairwise correlations using probes that have detection *P* value below 0.05 (Supplemental Figure 12). Based on hierarchical clustering, we removed 1 outlier sample from the liver (D000008) from further analysis. This sample comprised markedly lower genomic DNA mass compared with the other samples.

### EWAS.

EWAS was conducted in a combined group of young and aged RMs. As covariates in the multivariate regression models, we used time point, chronological age, and sex for the PBMCs. The models were fitted using R v.4.2 programming language and *limma* v3.50.3 package (90). The model formulas were the following: ~1 + Timepoint + Age + Sex for the basic model and ~1 + Timepoint + Age + Sex + CD4^+^ T cells for a model adjusted for absolute CD4^+^ T cell abundance in the blood. Limma’s *duplicateCorrelation* function was used to account for repeated measurements taken from the same individual with the blocking for animal ID variable that was used as a random effect. Empirical Bayes correction was applied to model fits. Limma least squares method was used (90). Limma’s contrasts.fit function was used to perform group comparisons. Contrasts used were the following: A versus B = A – B, EC versus B = EC – B, and LC versus B = LC – B, where B is baseline, A is acute, EC is early chronic, and LC is late chronic. *P* values were adjusted with Benjamini-Hochberg correction for multiple testing, and those with FDR *q* less than 0.05 were deemed significant.

Pathway analysis was conducted with IPA (QIAGEN) (47). We selected genes associated with CpGs that passed adjusted differential methylation *P* value of 0.05 and analyzed them against the gene content of the HorvathMammalMethylChip40 as a background.

### Clock analysis.

We applied 10 different epigenetic estimators of biological age based on DNAme data: primate clocks (DNAmAge.RhesusBloodTissue, DNAmAge.RhesusPanTissue, DNAmAge.PrimateClock), pan-mammalian clocks (UniversalClock3Pan, UniversalClock2Skin, UniversalClock3Skin, UniversalClock2Pan, UniversalClock2Blood, UniversalClock3Blood), and DNAmAge.PrimateRelativeAge (48, 91, 92). EAA was measured as a difference between EA and chronological age and modeled as a function of age status (young or aged) in all individuals (Clock-Age ~0 + Age + Status) and EA in the solid tissues was modeled as a function of dpi (when tissues were harvested at necropsy) and age in the group of young and aged adults using the following formula: lm = Clock ~1 + dpi + Age, data = *x*.

### Pathogenesis biomarker association with EA.

To identify biomarkers associated with EA, we used data from a set of key phenotypic measures linked to SIV/HIV pathogenesis representing major aspects of disease mechanisms (categories): CD4^+^ T cell population (in blood, SLNs, gut); viral loads (plasma viral loads, CA-vDNA, CA-vRNA); T cell activation, indicated by the fraction of CD4^+^ and CD8^+^ T cells expressing the marker of late activation CD25, the marker of cell proliferation Ki-67, and coexpressing CD38 and HLA-DR (in periphery: CD25^+^CD4^+^ and CD25^+^CD8^+^ T cells; periphery and gut: Ki-67^+^CD4^+^ and Ki-67^+^CD8^+^ T cells; HLA-DR^+^CD38^+^CD4^+^ and HLA-DR^+^CD38^+^CD8^+^ T cells); inflammation (CRP, IL-1B, IL-1RA, IL-2, IL-6, IL-12, IL-15, IL-17, IL-8, IP-10, and MIG); microbial translocation (sCD163 and sCD14); and coagulation (D-dimer).

We used Spearman’s rank correlation to identify biomarkers correlating with EAA (Clock-Age). For all preinfection samples, the dpi value was set to 0 and the dpi variable was scaled. Since dpi and age variables are inherently interconnected and self-explanatory, models examining the correlation between dpi and EA do not account for age differences between animals.

We conducted ANOVA to assess whether incorporating biomarkers of pathogenesis into the model yielded a significant enhancement in clock predictions. For the longitudinal PBMC data, we used the full model formula: UniversalClock3Blood ~Time point + Age + Sex + biomarker + (1 + Age | Animal ID). The null model formula serving as a baseline was UniversalClock3Blood ~Time point + Age + Sex + (1 + Age | Animal ID). By comparing these models, we aimed to determine the impact of including each individual biomarker of pathogenesis as a predictor of the accuracy of UniversalClock3Blood predictions, accounting for other variables such as time point, age, sex, and individual variations represented by Animal ID.

### Statistics.

To compare the dynamics of different pathogenesis variables throughout the follow-up period, we conducted exploratory analyses using the Mann-Whitney *U* test to compare baseline values and the AUC for the variables plotted in Figures 1–4. For the AUC (for variable log) analyses, we used the function integrate.xy from the package sfsmisc in R, and considered the maximal time for which we had data for all RMs: first 200 dpi for measurements in the periphery and first 100 days for measurements in lymph nodes or intestine. Additionally, to better dissect early and late changes, we used mixed-effects models to analyze the repeated measurements dynamics of these variables during 2 follow-up periods: (a) changes during acute infection (<18 days for most variables, except for cytokines in Figure 3, where these changes were faster: <10 days), and (b) the chronic period from 30 dpi onward. We used the package lmer in R for these analyses. This approach enabled us to use all measurements and analyze whether the acute changes were different between young and old RMs (testing for the interaction term) and whether the chronic period showed a difference between young and old RMs. The latter comparison can reveal differences between young and old RMs in the initial level (at 30 days), in slopes of change (interaction term), and if the interaction is not significant in overall slope (increase or decrease). We present the results in a table of *P* values for these comparisons (Supplemental Table 1). This being an exploratory analysis, we did not perform any post hoc correction for multiple comparisons. In the box-and-whisker plots, the bounds of the boxes represent the interquartile range (IQR), spanning from the 25th to the 75th percentile. The line within each box indicates the median (50th percentile). The whiskers extend to the most extreme data points that are within 1.5 times the IQR from the lower and upper quartiles. Data points are shown.

For the Wilcoxon and ANOVA tests, we used 2-sided tests. For all epigenetic biomarker analyses (EA and EAA), we used *P* values and reported statistically significant differences if the *P* value was less than 0.05.

### Study approval.

Animal procedures and sample collections were reviewed and approved by the University of Pittsburgh IACUC (protocol 15055919) and were in compliance with the NIH *Guide for the Care and Use of Laboratory Animals* (93). Animal procedures met or exceeded all standards of the Public Health Service’s Policy on the Humane Care and Use of Laboratory Animals (94). The animals were fed and housed according to regulations set forth by the NIH *Guide for the Care and Use of Laboratory Animals* and the Animal Welfare Act (93).

### Data availability.

Our tissue and PBMC DNAme datasets were deposited in the NCBI’s GEO under accession numbers GSE226858 and GSE226899, respectively. Biomarker levels, as well as values for all data points shown in graphs, are reported in the 2 Supporting Data Values files.

## Author contributions

AJJ acquired and analyzed data, performed in-depth/comprehensive analysis of biomarker data, designed research studies, and wrote the manuscript. RS acquired and analyzed data, performed preliminary analysis of pathogenesis biomarkers, and wrote the manuscript. SS, CX, JG, MM, RTB, PS, TH, EBC, BBP, KN, ST, HA, and DM acquired and analyzed data. RMB analyzed data. CA and IP provided project management, designed research studies, analyzed data, and wrote the manuscript.

## Supplementary Material

Supplemental data

Supplemental tables 1-5

Supporting data values

## Figures and Tables

**Table 1:**
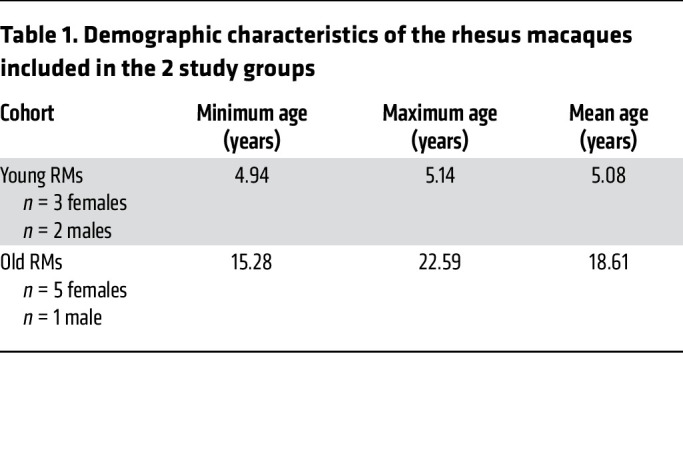
Demographic characteristics of the rhesus macaques included in the 2 study groups

**Table 2:**
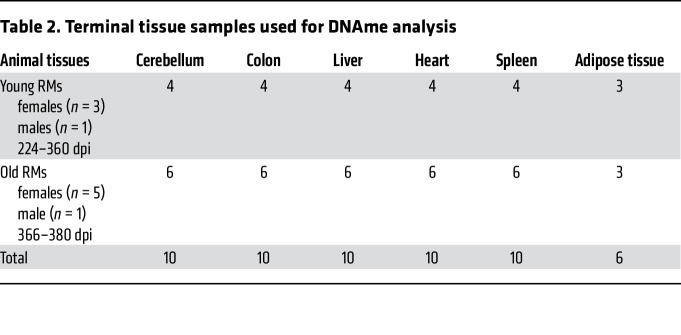
Terminal tissue samples used for DNAme analysis

**Table 3:**
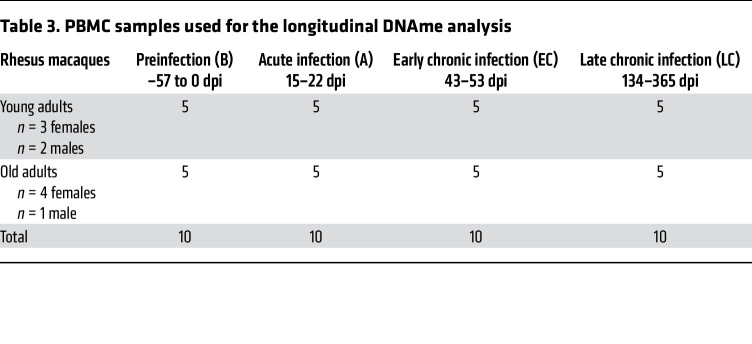
PBMC samples used for the longitudinal DNAme analysis

## Version 1 07/15/2025

Electronic publication
