# Supplementary material for: Pathogenic SIV infection is associated with acceleration of epigenetic age in rhesus macaques
Source: J Clin Invest. 2025 Jul 15;135(14):e189574. doi: 10.1172/JCI189574 (PMC12259264; doi:10.1172/JCI189574)
Supplement: Supplemental data [file jci-135-189574-s037.pdf]

Pathogenic simian immunodeficiency virus infection is associated with acceleration of epigenetic age in Rhesus macaques

----

Running title: **Epigenetic aging and SIV infection**

Anna J Jasinska,[1,2¶] Ranjit Sivanandham,[1,2¶] Sindhuja Sivanandham,[1,2] Cuiling Xu,[1,2] Juozas Gordevicius,[3] Milda Milciute,[3] Robert T Brooke,[3] Paola Sette,[1,2] Tianyu He,[2] Egidio Brocca-Cofano,[1] Benjamin B. Policicchio,[5] Krishna Nayak,[2] Saharsh Talwar, [2] Haritha Annapureddy,[2] Dongzhu Ma,[1,2] Ruy Ribeiro[4], Cristian Apetrei,[1,5] and Ivona Pandrea[2,5*]

Supplemental Information 1

Content

1. Functional enrichment analysis of the peripheral blood mononuclear cell (PBMC) epigenome-wide association studies (EWAS) signals associated with SIV infection
2. Genes associated with DNAme in both acute (A) and late chronic (LC) stages of SIV infection
3. A comparative ingenuity pathway analysis (IPA) of the EWAS signals
4. EWAS analysis adjusted for the $CD4^+$ T-cell abundance

1. Functional enrichment analysis of the peripheral blood mononuclear cell (PBMC) epigenome-wide association studies (EWAS) signals associated with SIV infection. Longitudinal DNAme profiles were used for EWAS analysis to identify CpG sites differentially methylated during different stages of SIV infection (acute–A, early chronic–EC, late chronic–LC) compared to baseline (B). We focused on the CpGs significantly associated with each stage of infection that were localized within or in the proximity of known genes.

***1a. Differential methylation during the acute (A) SIV infection*.** The top differentially hypomethylated CpGs during the A stage were associated with the *SPRED2* gene, a member of the Sprouty/SPRED family of proteins that regulate growth factor-induced activation of the MAP kinase cascade; *TNRC6B* gene, a gene expression regulator; and *ASXL1* gene, a member of the Polycomb group of proteins involved in developmental regulation.

The top hypermethylated CpGs in the A stage were proximal to the *ZBTB7B* gene (AKA *ThPOK*, *ZFP67*), which codes a transcriptional master regulator of (critical regulator of cell fate determination) the T-cell commitment (1-3), *ILF3* (interleukin enhancer binding factor 3), which is a negative regulator of dendritic cell maturation and interferon (IFN) responses (4); *PDCD1* (AKA PD-1), which codes the programmed cell death protein 1, a protein of multifaceted functions that acts as an immune-inhibitory receptor expressed in activated T cells, regulating T-cell functions, and involved in regulating autoimmunity and anti-tumor immunity.

The most significant longitudinal changes in DNAme in the PBMCs were associated with the *SPRED2* and *ZBTB7B* genes, which, respectively, were top hypomethylated and hypermethylated throughout the follow-up. *SPRED2* participates in negative regulation of innate immune responses as an inhibitor of IFN-γ production by $CD4^+$ and $CD8^+$ T-cells and an inhibitor of ERK-MAPK pathway (5). Its deficiency protects

from polymicrobial sepsis *via* increased activation of the ERK/MAPK pathway and subsequent increase in innate immune responses in the mouse model (6). *ZBTB7B* is a key differentiation factor during positive selection mediating the $CD4^+$ T-cell commitment and preventing $CD8^+$ T-cell commitment by inhibiting the expression of cytotoxic differentiation markers (2, 7). Whether the hypermethylation of ZBTB7B influences changes in the $CD4^+$/$CD8^+$ T-cell equilibrium occurring through the infections needs to be experimentally tested.

1b. Differential methylation during the early chronic (EC) SIV infection. The gene regions with top significant CpG sites differentially methylated in the LC stage were the same as for the A stage: *SPRED2* (hypomethylated) and *ZBTB7B* (hypermethylated). Other top hypomethylated genes were *ZEB2* (a transcription factor and a regulator of chromatin remodeling, development and age-associated B-cell differentiation (8)), *CRIM1*, which, via binding growth factors, contributes to motor neuron differentiation, angiogenesis, and muscle aging (9) and EIF4E3 (as in late chronic infection). Top hypermethylated genes included *ILF3* (as in A) and *DDX5* (DEAD box helicase 5), which is an RNA helicase involved in alterations of RNA structure during translation initiation, splicing, and ribosome and spliceosome assembly.

1c. Differential methylation during the late chronic (LC) SIV infection. The top genes associated with hypomethylated CpG sites in the LC stage were *SPRED2* (also the top gene in A and EC), *ZEB2* (also a top gene in EC), *CRIM1*, which *via* binding growth factors contributes to motor neuron differentiation, angiogenesis, and muscle aging (9), and *EIF4E3* (as in EC). The top hypermethylated genes were *LEF1* (a member of the T-cell factor (TCF)/LEF1 family transcription factors involved in the Wnt/β-catenin signaling pathway (10) and repressing $CD4^+$ T-cell lineage genes, thus promoting $CD8^+$ T-cell identity (11), *ZBTB7B* (also hypermethylated in A and EC), and *MBTPS1*, which is

a transcription factor involved in the cholesterol metabolism of the HIV-1 exposed seronegative individuals (12).

Altogether, the infection influenced methylation of CpGs associated with genes involved HIV/SIV pathogenesis (latency, T-cell differentiation) and related with HANA conditions (e.g., atherosclerosis).

Supplemental Information Table 1. Genes around the top significant EWAS hits

Top Genes	Acute infection	Early chronic infection	Late chronic infection
Hypomethylation	SPRED2<br>TNRC6B<br>ASXL1	SPRED2<br>ZEB2<br>EIF4E3	SPRED2<br>ZEB2<br>CRIM1
Hypermethylation	ZBTB7B<br>ILF3<br>PDCD1 (PD-1)	ZBTB7B<br>ILF3<br>DDX5	ZBTB7B<br>LEF1<br>MBTPS1

2. Genes associated with DNAme in both A and LC stages of infection. The acute and late chronic stages of SIV infection showed concordant changes in methylation at the CpG sites associated 541 genes (hypomethylation) and 161 (hypermethylation). Functional analysis of these genes with David using the entire mammalian chip content as a background (13) showed main functional categories enriched among the genes containing or localized nearby the sites hypermethylated or hypomethylated in the A and LC stages (Benjamini-Hochberg corrected p-value <0.05). Hypermethylated genes were significantly enriched for transcriptional regulation, transcription, and UBL conjugation (Supplemental Tables 2, 3). The host ubiquitin-conjugating system regulates the localization and abundance of viral proteins, while HIV also exploits the UBL conjugation system at all stages of viral life cycle (14). Hypermethylated genes were highly significantly enriched for terms related to either localization in the nuclear compartment

and/or transcription regulation by polymerase RNA II (Supplemental Tables 4, 5). It is consistent with the fact that both HIV and SIV fully relay on the host RNA polymerase II to transcribe their genome and produce viral particles (13).

3. **A comparative IPA pathway analysis of the EWAS signals.** To identify groups of biological functions associated with different stages of the SIV infection, we conducted a functional enrichment analysis of the genes associated with the CpGs differentially methylated at key timepoints of SIV infection in the PBMCs using IPA (QIAGEN Inc., https://www.qiagenbioinformatics.com/products/ingenuity-pathway-analysis) (15) (Supplemental Figures 8-9).

3a. Pathways differentially methylated in the PBMCs during the infection. The acute stage of SIV infection in the PBMCs was associated with differential methylation of the genes in the aryl hydrocarbon receptor (ARH) signaling pathway (Supplemental Figure 8A). The ARH signaling pathway is activated through oncometabolite kynurenine and is associated with an increased tryptophan catabolism by IDO metabolizing leading to decrease of tryptophan levels and elevated levels of a kynurenine and other downstream metabolites, which modulate immune responses and inflammation. The ARH regulates immune responses in autoimmunity, infections and tumors (16) and has a multifaceted effect on adaptive immunity modulating T-cell differentiation and function. AHR activation promotes HIV infection and lymphocyte activation/virus reactivation (17); conversely, it causes a block of HIV-1 replication in macrophages (18).

(i) Role of BRCA1 in the DNA damage response pathway. The DNA damage response pathway was the second most significant pathway associated with changes in methylation profiles in the PBMCs during the acute SIV infection. DNA damage repair

mechanisms, such as those engaging BRCA1 functions, are involved in various steps of the viral life cycle, including integration and Vpr-induced G2/M arrest in the $CD4^+$ T-cells; moreover, BRCA1 acts as an enhancer of HIV-1 transcription in the infected T-cells (19, 20).

(ii) The activin inhibin signaling pathway was the next PBMC pathway associated with the acute SIV infection: Activin A (the most widely studied molecule of this group), is a multifunctional cytokine, which is a member of the transforming growth factor (TGF)–β family that plays a crucial role in wound repair and in fetal tolerance, is a mediator in inflammation, inducer of the directional migration of immature myeloid dendritic cells, a regulator of hematopoiesis, as well as a participant on the pathophysiology of cancer and fibrotic syndromes (21). Lipopolysaccharide elicit activin A production from myeloid DCs (22), however, unlike multiple other cytokines, which undergo rapid induction during the first few days post HIV-1 infection (23, 24) activins are not activated in the cytokine/chemokine storm (22).

IPA pointed to the association of acute SIV infection with a cluster related to liver tumorigenesis (liver tumor, liver carcinoma, liver cancer) and familial congenital heart disease; meanwhile, early chronic SIV infection was associated with familial dilated cardiomyopathy (Supplemental Figure 8B).

The top enriched disease categories were associated with both A and LC infection (Supplemental Figure 9A). They included oral oncological terms (oral tumor, oral squamous carcinoma, oral cancer) concordantly with an increased risk of head and neck squamous cell carcinoma, including squamous cell carcinoma, and elevated risk of HPV infection (~2-fold) (25, 26). Also, T-cell malignant neoplasms category was shared between A and LC stage concordantly with an increased risk of T cell lymphomas due to HIV infection and resultant deregulation and depletion of T cells (27).

The top significantly enriched clusters of molecular and cellular functions were associated with all three stages of the infection (A, EC, LC), yet stronger association was observed for A and LC stages (Supplemental Figure 9B). They were related to transcription regulation (transcription, expression of RNA, activation of DNA endogenous promoter) and cell cycle regulation (arrest of cell cycle progression, differentiation of progenitor and stem cells, cell proliferation, cell cycle progression) highlighting the impact of SIV infection on the host gene expression machinery and deregulation of cell cycle. Other cellular and molecular categories highly associated with infection, particularly A and LC stages, included hematopoiesis of mononuclear leukocytes, leukopoiesis, T cell depletion and differentiation that mirrors HIV-related disruption of leukocyte development (28).

Whether the observed alterations in DNA methylation affect gene expression needs to be verified through quantitative gene expression, metabolic and proteomic analyses. Importantly, some of the pathways associated with infection, particularly those linked to oncogenesis, are potentially targetable using small molecules, for example, AHR signaling (29).

4. EWAS analysis adjusted for the CD4$^+$ T-cell abundance

CD4$^+$ T-cell loss is a hallmark of HIV/SIV infection and progression. When we adjusted EWAS analysis for the absolute counts of CD4$^+$ T-cells, we observed fewer significant signals. In the acute stage, 66 CpGs (associated with 48 genes) were hypomethylated, while 66 CpGs (associated with 53 genes) were hypermethylated. In the early chronic stage, 9 CpGs (associated with 7 genes) were hypomethylated, while 47 CpGs (associated with 28 genes) were hypermethylated. In the late chronic stage, 17 CpGs (associated with 13 genes) were hypomethylated, while 98 CpGs (associated with 41 genes) were hypermethylated. Despite a smaller number of stage-associated CpGs,

hypomethylation of *SPRED2* and hypermethylation of *ZBTB7B*, ZEB2, *ILF3*, and *DDX* were observed across three stages of the infection.

References

1. Naito T, and Taniuchi I. The network of transcription factors that underlie the CD4 versus CD8 lineage decision. *Int Immunol.* 2010;22(10):791-6.
2. He X, He X, Dave VP, Zhang Y, Hua X, Nicolas E, et al. The zinc finger transcription factor Th-POK regulates CD4 versus CD8 T-cell lineage commitment. *Nature.* 2005;433(7028):826-33.
3. He X, Park K, and Kappes DJ. The role of ThPOK in control of CD4/CD8 lineage commitment. *Annu Rev Immunol.* 2010;28:295-320.
4. Nazitto R, Amon LM, Mast FD, Aitchison JD, Aderem A, Johnson JS, et al. ILF3 Is a Negative Transcriptional Regulator of Innate Immune Responses and Myeloid Dendritic Cell Maturation. *J Immunol.* 2021;206(12):2949-65.
5. Sun C, Fujisawa M, Ohara T, Liu Q, Cao C, Yang X, et al. Spred2 controls the severity of Concanavalin A-induced liver damage by limiting interferon-gamma production by CD4+ and CD8+ T cells. *J Advert Res.* 2022;35:71-86.
6. Itakura J, Sato M, Ito T, Mino M, Fushimi S, Takahashi S, et al. Spred2-deficiecy Protects Mice from Polymicrobial Septic Peritonitis by Enhancing Inflammation and Bacterial Clearance. *Sci Rep.* 2017;7(1):12833.
7. Sun G, Liu X, Mercado P, Jenkinson SR, Kypriotou M, Feigenbaum L, et al. The zinc finger protein cKrox directs CD4 lineage differentiation during intrathymic T cell positive selection. *Nat Immunol.* 2005;6(4):373-81.
8. ASXL1 mutation accelerates atherosclerosis by promoting activation of myeloid cells. *Nat Cardiovasc Res.* 2024;3(12):1387-8.
9. Tumasian RA, 3rd, Harish A, Kundu G, Yang J-H, Ubaida-Mohien C, Gonzalez-Freire M, et al. Skeletal muscle transcriptome in healthy aging. *Nat Commun.* 2021;12(1):2014.
10. Santiago L, Daniels G, Wang D, Deng F-M, and Lee P. Wnt signaling pathway protein LEF1 in cancer, as a biomarker for prognosis and a target for treatment. *Am J Cancer Res.* 2017;7(6):1389-406.
11. Xing S, Li F, Zeng Z, Zhao Y, Yu S, Shan Q, et al. Tcf1 and Lef1 transcription factors establish CD8(+) T cell identity through intrinsic HDAC activity. *Nat Immunol.* 2016;17(6):695-703.
12. Saulle I, Ibba SV, Vittori C, Fenizia C, Mercurio V, Vichi F, et al. Sterol metabolism modulates susceptibility to HIV-1 Infection. *AIDS.* 2020;34(11):1593-602.
13. Huang da W, Sherman BT, and Lempicki RA. Systematic and integrative analysis of large gene lists using DAVID bioinformatics resources. *Nat Protoc.* 2009;4(1):44-57.

14. Proulx J, Borgmann K, and Park IW. Post-translational modifications inducing proteasomal degradation to counter HIV-1 infection. *Virus Res.* 2020;289:198142.
15. Krämer A, Green J, Pollard J, and Tugendreich S. Causal analysis approaches in Ingenuity Pathway Analysis. *Bioinformatics.* 2014;30(4):523-30.
16. Dean JW, Helm EY, Fu Z, Xiong L, Sun N, Oliff KN, et al. The aryl hydrocarbon receptor cell intrinsically promotes resident memory CD8(+) T cell differentiation and function. *Cell Rep.* 2023;42(1):111963.
17. Zhou Y-H, Sun L, Chen J, Sun W-W, Ma L, Han Y, et al. Tryptophan Metabolism Activates Aryl Hydrocarbon Receptor-Mediated Pathway To Promote HIV-1 Infection and Reactivation. *MBio.* 2019;10(6).
18. Kueck T, Cassella E, Holler J, Kim B, and Bieniasz PD. The aryl hydrocarbon receptor and interferon gamma generate antiviral states via transcriptional repression. *Elife.* 2018;7, 10.7554/eLife.38867.
19. Andersen JL, Zimmerman ES, DeHart JL, Murala S, Ardon O, Blackett J, et al. ATR and GADD45alpha mediate HIV-1 Vpr-induced apoptosis. *Cell Death Differ.* 2005;12(4):326-34.
20. Guendel I, Meltzer BW, Baer A, Dever SM, Valerie K, Guo J, et al. BRCA1 functions as a novel transcriptional cofactor in HIV-1 infection. *Virol J.* 2015;12:40.
21. Morianos I, Papadopoulou G, Semitekolou M, and Xanthou G. Activin-A in the regulation of immunity in health and disease. *J Autoimmun.* 2019;104:102314.
22. Dickinson M, Kliszczak AE, Giannoulatou E, Peppa D, Pellegrino P, Williams I, et al. Dynamics of Transforming Growth Factor (TGF)-beta Superfamily Cytokine Induction During HIV-1 Infection Are Distinct From Other Innate Cytokines. *Front Immunol.* 2020;11:596841.
23. von Sydow M, Sönnerborg A, Gaines H, and Strannegård O. Interferon-alpha and tumor necrosis factor-alpha in serum of patients in various stages of HIV-1 infection. *AIDS Res Hum Retroviruses.* 1991;7(4):375-80.
24. Stacey AR, Norris PJ, Qin L, Haygreen EA, Taylor E, Heitman J, et al. Induction of a striking systemic cytokine cascade prior to peak viremia in acute human immunodeficiency virus type 1 infection, in contrast to more modest and delayed responses in acute hepatitis B and C virus infections. *J Virol.* 2009;83(8):3719-33.
25. McLemore MS, Haigentz M, Jr., Smith RV, Nuovo GJ, Alos L, Cardesa A, et al. Head and neck squamous cell carcinomas in HIV-positive patients: a preliminary investigation of viral associations. *Head Neck Pathol.* 2010;4(2):97-105.
26. Beachler DC, Weber KM, Margolick JB, Strickler HD, Cranston RD, Burk RD, et al. Risk factors for oral HPV infection among a high prevalence population of HIV-positive and at-risk HIV-negative adults. *Cancer Epidemiol Biomarkers Prev.* 2012;21(1):122-33.
27. Lurain K, Ramaswami R, and Yarchoan R. The role of viruses in HIV-associated lymphomas. *Semin Hematol.* 2022;59(4):183-91.
28. Tsukamoto T. Hematopoietic Stem/Progenitor Cells and the Pathogenesis of HIV/AIDS. *Front Cell Infect Microbiol.* 2020;10:60.

29. Faber SC, Lahoti TS, Taylor ER, Lewis L, Sapiro JM, Toledo Sales V, et al. Current Therapeutic Landscape and Safety Roadmap for Targeting the Aryl Hydrocarbon Receptor in Inflammatory Gastrointestinal Indications. *Cells.* 2022;11(10).

Pathogenic SIVmac Infection of Rhesus Macaques Is Associated with Acceleration of Epigenetic Age

----

Running title: **Epigenetic aging and SIV infection**

Anna J Jasinska,[1,2¶] Ranjit Sivanandham,[1,2¶] Sindhuja Sivanandham,[1,2] Cuiling Xu,[1,2] Juozas Gordevicius,[3] Milda Milciute,[3] Robert T Brooke,[3] Paola Sette,[1,2] Tianyu He,[2] Egidio Brocca-Cofano,[1] Benjamin B. Policicchio,[4] Krishna Nayak,[2] Saharsh Talwar, [2] Haritha Annapureddy,[2] Dongzhu Ma,[1,2] Ruy Ribeiro, [4] Cristian Apetrei,[1,5] and Ivona Pandrea[2,5*]

SUPPLEMENTAL FIGURES 1-13

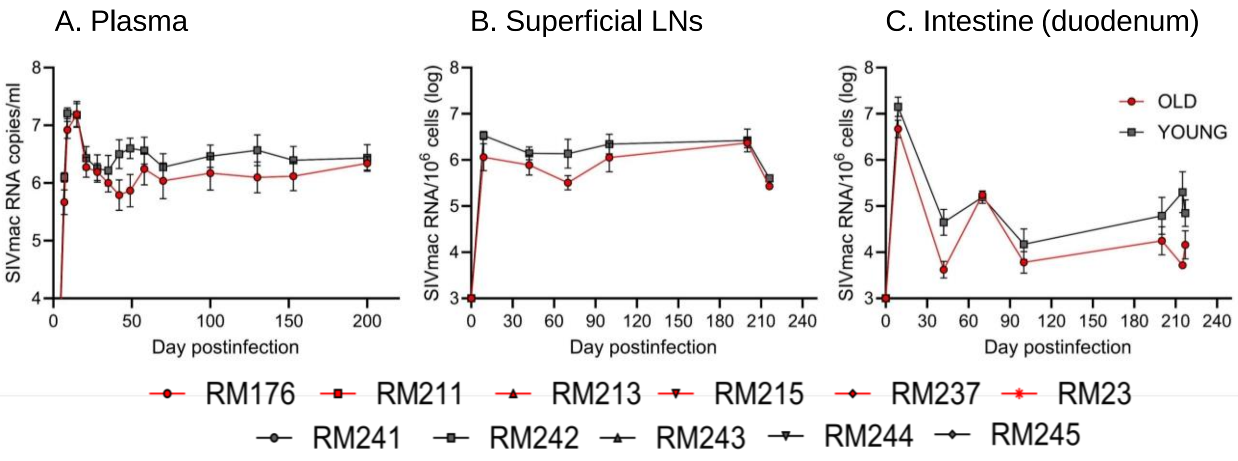

Supplemental Figure 1. Dynamics of pVLs (vRNA copies/ml) (A) and cell-associated viral RNA (CA-vRNA) (copies/10^6 cells) in the superficial lymph nodes (LNs) (B) and duodenum (C). Geometrical means and standard errors of the means are shown for old (red) and young (black) RMs

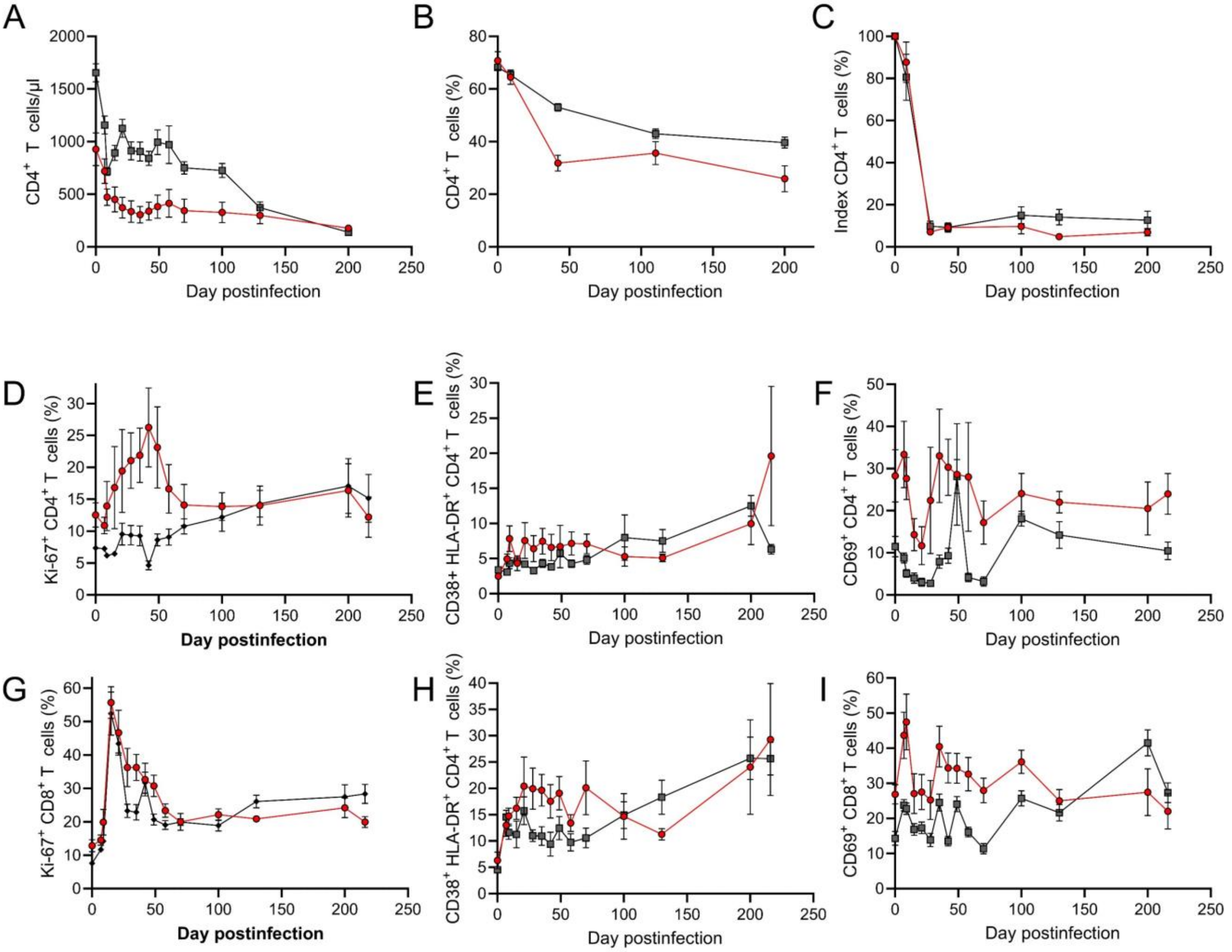

Supplemental Figure 2. Longitudinal changes in CD4$^+$ and CD8$^+$ T cell profiles. Dynamics of CD4$^+$ T cell counts in circulation (A), superficial lymph nodes (SLNs) (B), and gut (C). Changes in T lymphocyte proliferation assessed by measuring the expression of the fraction of circulating CD4$^+$ T cells (D) and CD8$^+$ T cells (G) expressing Ki-67. Dynamics of the expression of HLA-DR and CD38 on circulating CD4$^+$ T cells (E) and CD8$^+$ T cells (H). Dynamics of the expression of the early immunoactivity biomarker CD69 assessed on circulating CD4$^+$ T cells (F) and CD8$^+$ T cells (I). Averages and standard errors of the means are shown for old (red) and young (black) RMs

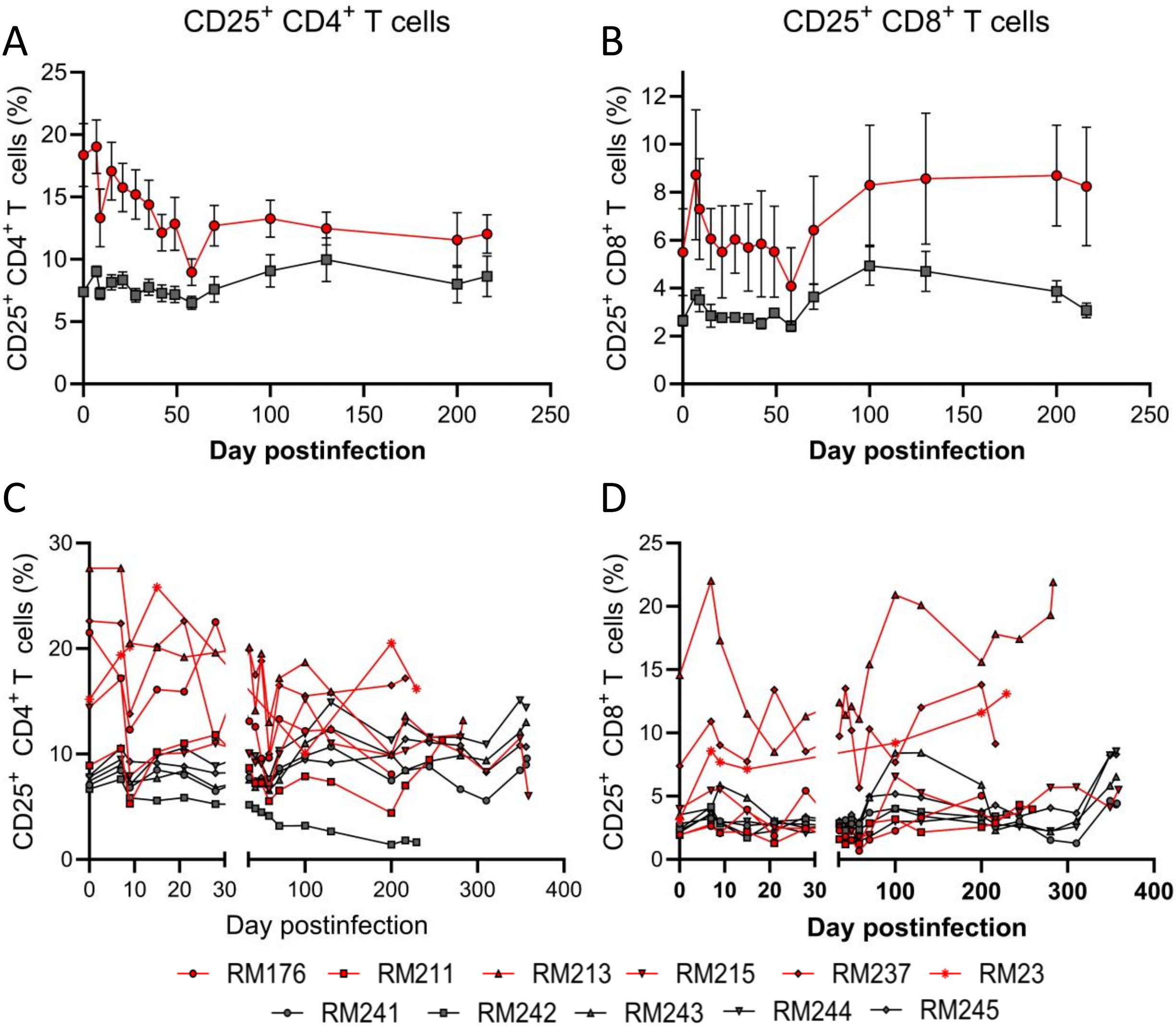

Supplemental Figure 3. Dynamics of the expression of CD25 on circulating $CD4^+$ T cells (A,C) and $CD8^+$ T cells (B,D). Averages and standard errors of the means are shown for old (red) and young (black) RMs (A,B), as well as individual dynamics (C,D)

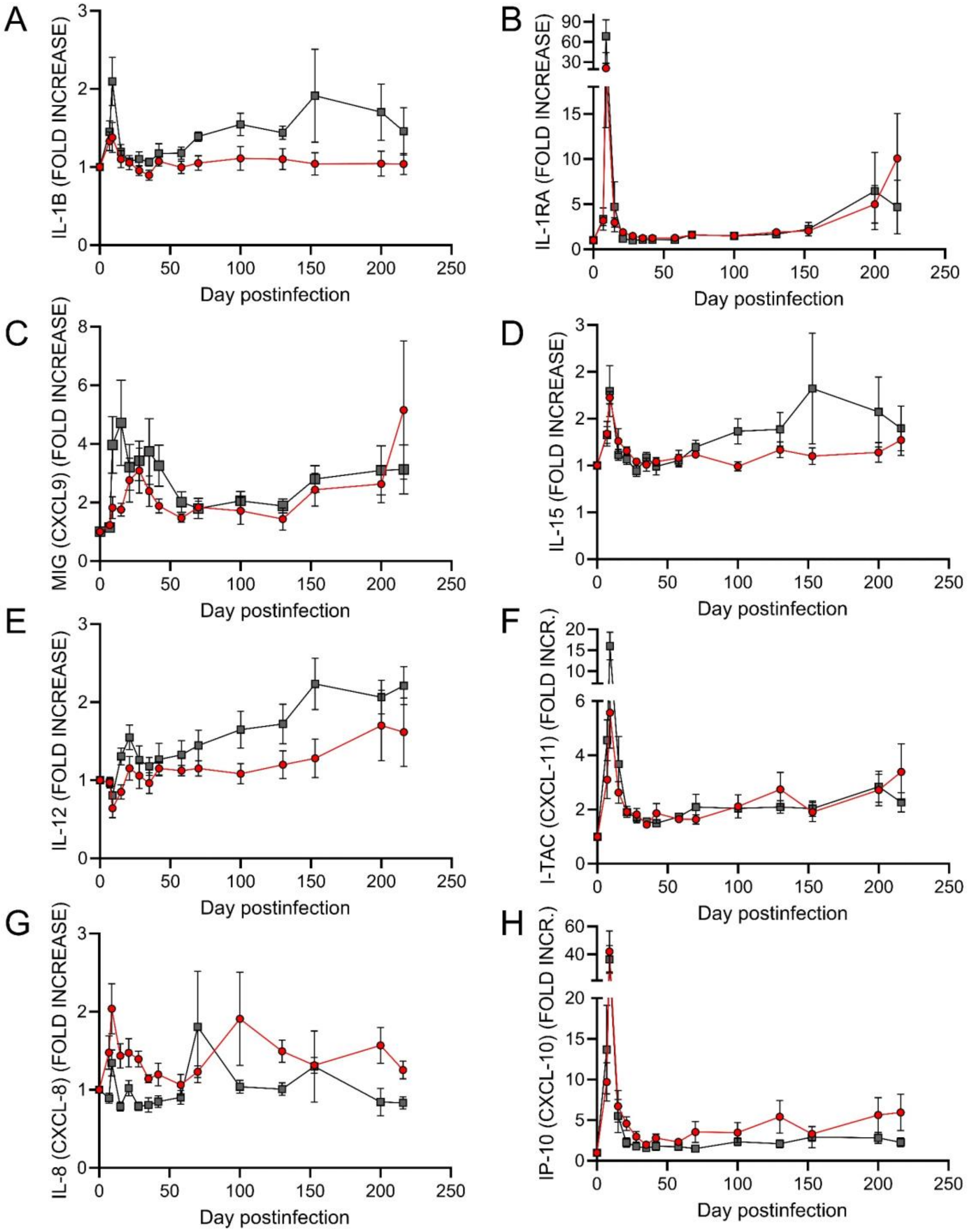

Supplemental Figure 4. Changes in the plasma levels of inflammatory molecules in SIVmac239-infected old (red) and young (black) rhesus macaques: IL-1B (A), IL-1RA (B), CXCL-9 (MIG) (C), IL-15 (D), IL-12 (E), CXCL-11 (I-TAC) (F), CXCL-8 (IL-8) (G), and CXCL-10 (IP-10) (H). Averages and standard errors of the means are shown for old (red) and young (black) RMs

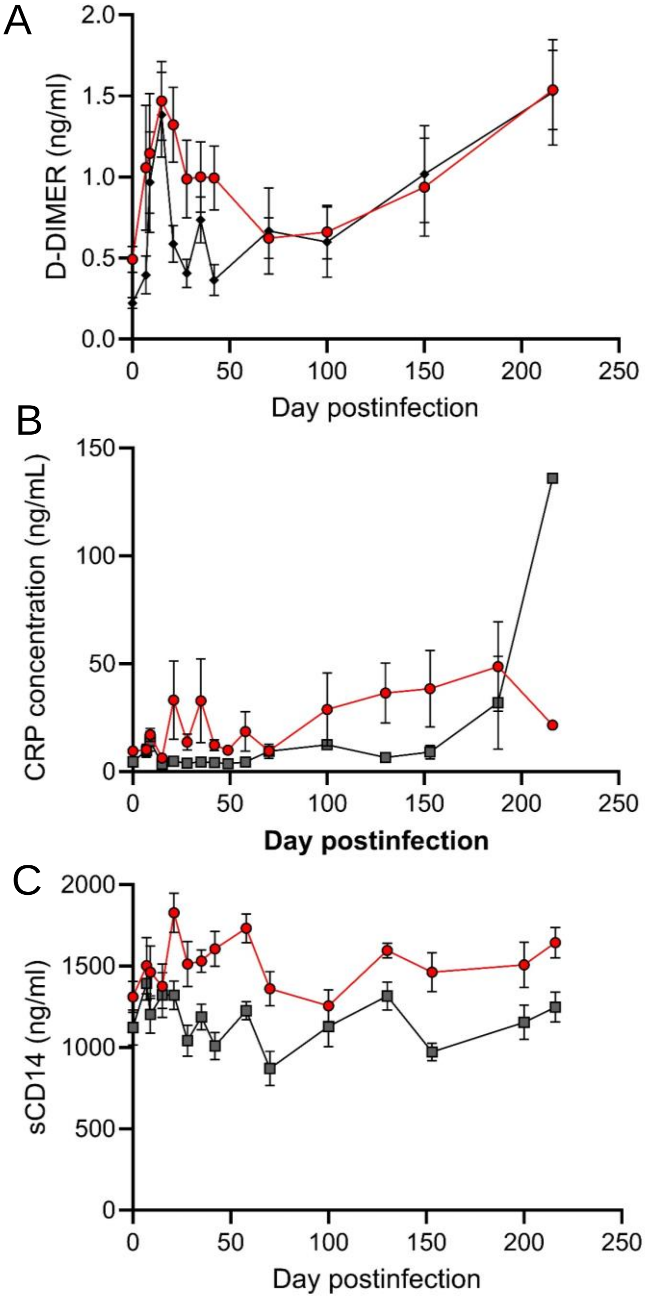

Supplemental Figure 5. Changes in the levels of biomarkers that were reported to be predictive for HIV disease progression and death in SIVmac-infected old (red) and young (black) RMS: D-dimer (A); C-reactive protein (CRP) (B); and sCD14 (C). Averages and standard errors of the means are shown for old (red) and young (black) RMs

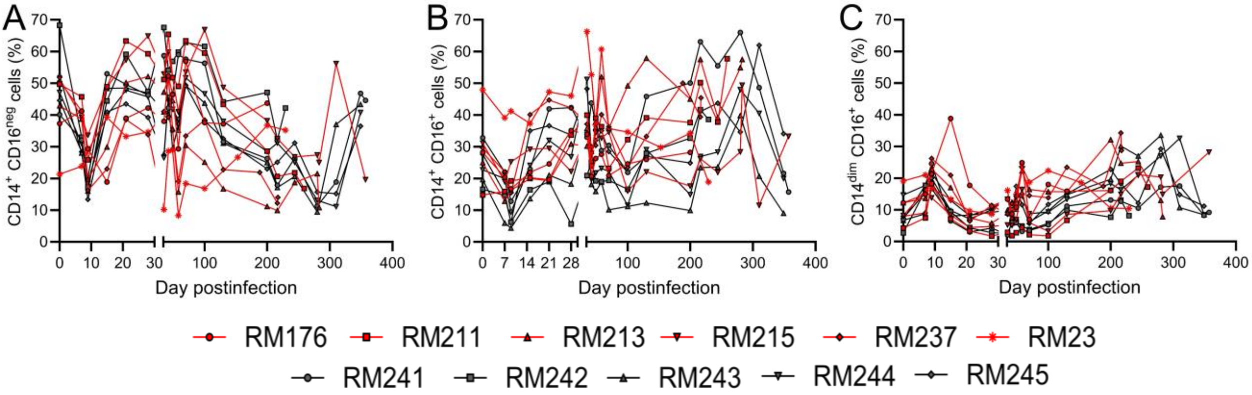

Supplemental Figure 6. Changes in the levels of major monocyte populations: classical ($CD14^{++}CD16^{neg}$) (A); intermediate ($CD14^{+}CD16^{+}$) (B); and nonclassical ($CD14^{dim}CD16^{+}$) (C) monocytes in old (in red) and young RMs (in black)

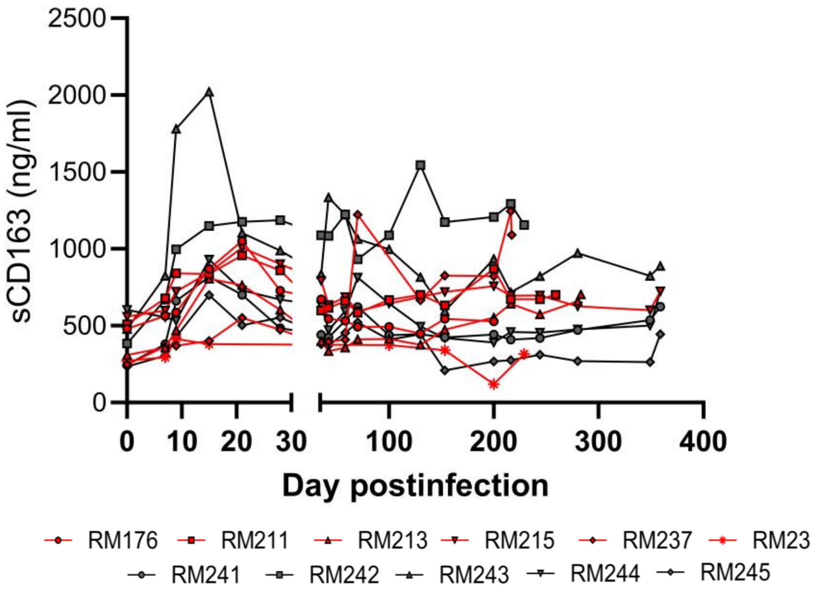

Supplemental Figure 7. Changes in the plasma levels of the macrophage activation marker sCD163 in old (red) and young (black) RMs.

A. Canonical Pathways in the PBMCs

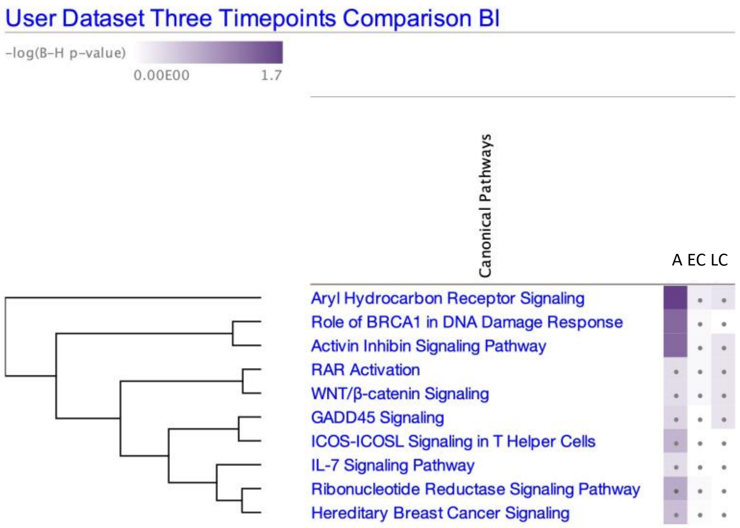

B. Toxicology Pathways in the PBMCs

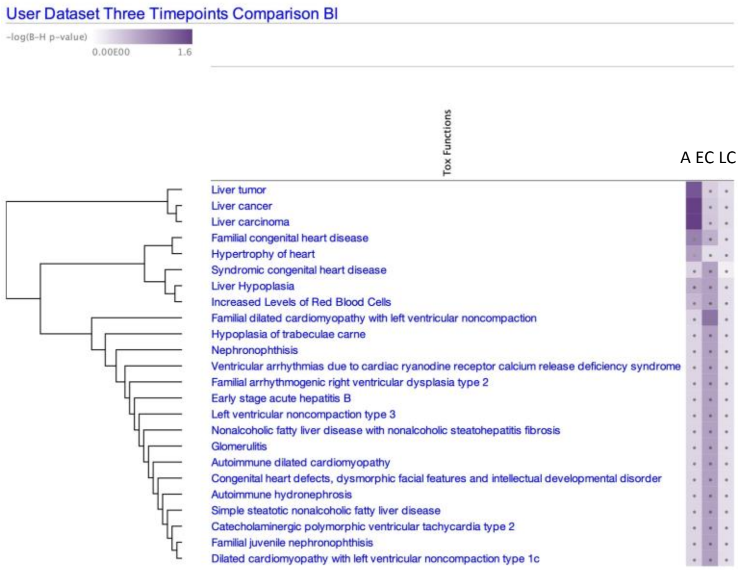

Supplemental Figure 8. Top IPA pathways associated with the course of infection in PBMCs: canonical pathways (A) and toxicological pathways (B). The pathway heat maps are based on Benjamini–Hochberg-corrected p-values from a right-tailed Fisher's exact test indicated by the intensity of the white-to-purple gradient. acute infection–A, early chronic infection–EC, late chronic infection–LC

A. Disease and Disorder in the PBMCs

B. Molecular and Cellular Functions in the PBMCs

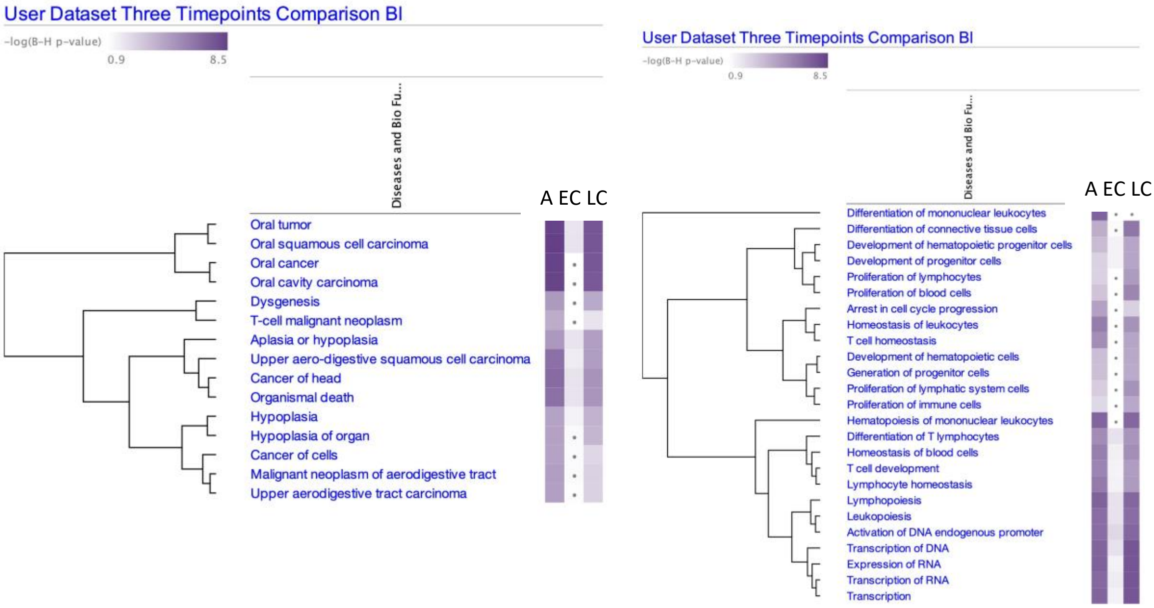

Supplemental Figure 9. Top ingenuity pathway analysis (IPA) associated with the course of infection in PBMCs: diseases (A) and molecular functions (B). The pathway heat maps are based on Benjamini–Hochberg-corrected p-values from a right-tailed Fisher's exact test indicated by the intensity of the white-to-purple gradient. acute infection–A, early chronic infection–EC, late chronic infection–LC

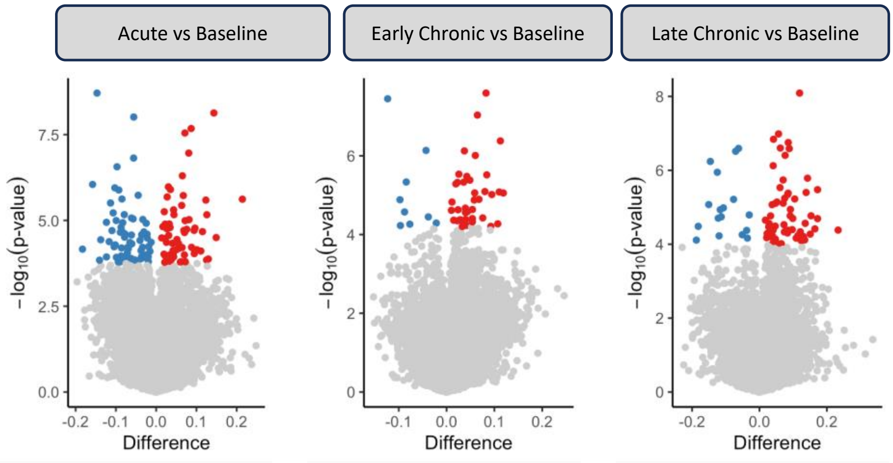

Contrast	Differentially methylated CpGs FDR q<0.05 Benjamini -Hochberg	Directionality of CpG methylation number of differentially methylated CpGs	
		Hypermethylation	Hypomethylation
EWAS for stage of SIV infection compared to the baseline in the PBMCs adjusted for CD4⁺ T-cell abundance			
A vs B	132	66	66
EC vs B	47	9	47
LC vs B	76	17	59

Supplemental Figure 10. Longitudinal DNAme analysis of PBMCs adjusted for absolute CD4$^+$ T-cell counts in the blood. (A) Epigenome-wide association study (EWAS) analysis of days postinfection (dpi) in PBMCs adjusted for peripheral CD4$^+$ T-cell levels, based on the entire methylation array. The volcano plots display the −log10 (p-values) and the directionality of association between CpG sites and infection stages (Acute-A, Early Chronic-EC, and Late Chronic-LC) are compared to the preinfection baseline (B): A *vs*. B (left panel), EC *vs*. B (central panel), and LC vs. B (right panel). Each dot represents a specific DNAme site. Significantly associated CpG sites (q<0.05) are shown in blue for hypomethylation and in red for hypermethylation, while nonsignificant CpGs are shown in gray. The horizontal axis represents the mean methylation change (i.e., the difference between group means), and the vertical axis represents −log10 (p-values). B-H: Benjamini–Hochberg correction; DMP: differentially methylated positions.

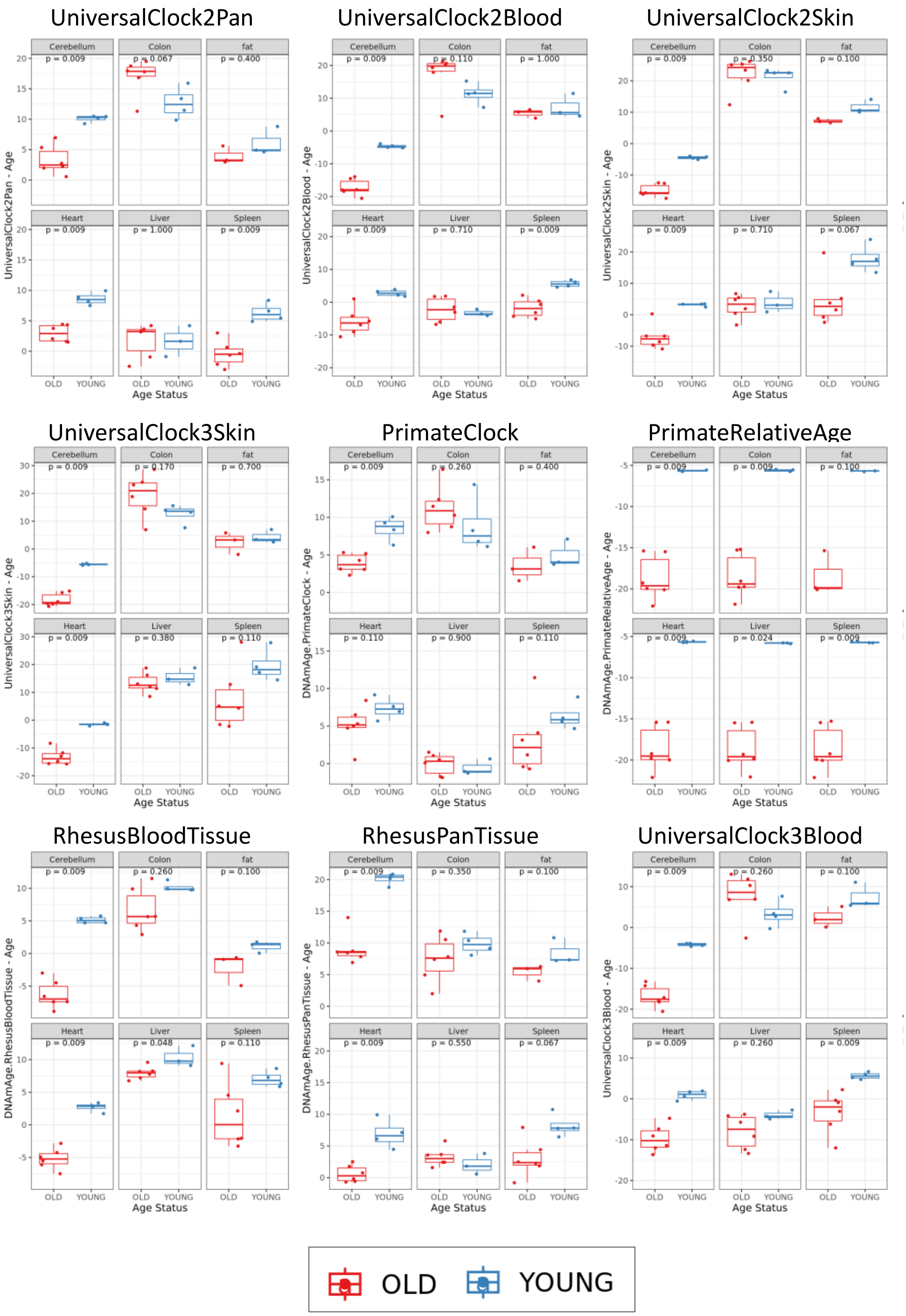

Supplemental Figure 11. Epigenetic age acceleration (EAA) in six tissue types in young (blue) and old (red) individuals during the late chronic SIVmac infection stage based on nine DNAme-based epigenetic clocks. The differences in EAA between the young (blue) and old (red) individuals were assessed using a two-sided Wilcoxon test.

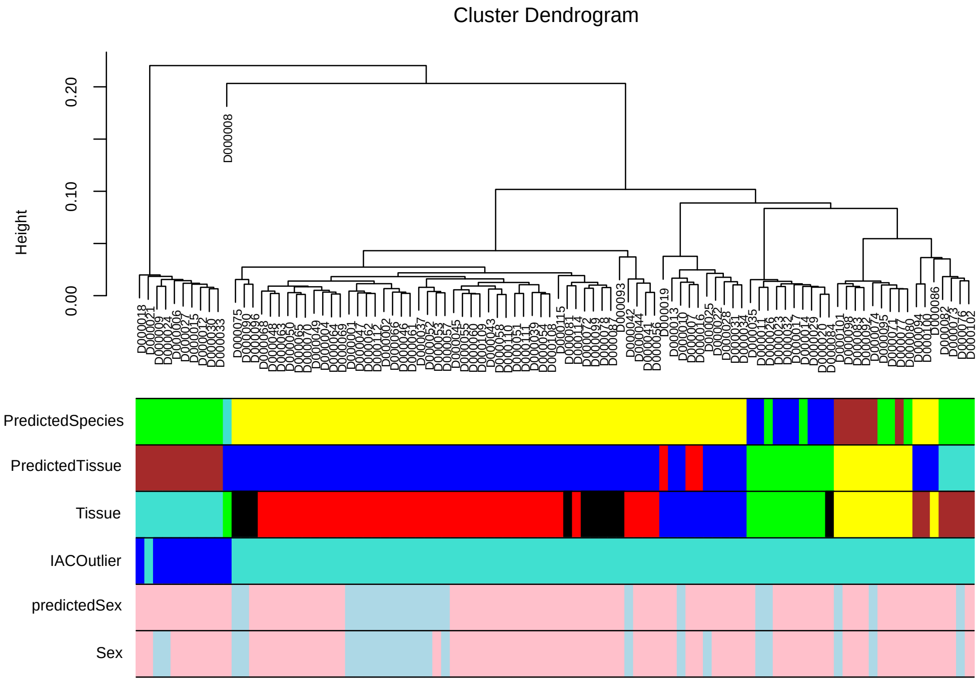

Supplemental Figure 12. Hierarchical clustering based on the pairwise correlations of the probes with the detection p-value<0.05. In the clustering dendrogram the height of a branch indicates dissimilarity of underlying samples to the rest. To assess intersample correlation, a Pearson correlation matrix was computed using the beta values with pairwise complete observations. A hierarchical clustering analysis was performed on the dissimilarity matrix (1 - corSample) using the agglomerative clustering method with average linkage (method = "a"). Tissue panel coloring key: cerebellum – turquoise, PBMCs – red, spleen – black, colon – blue, heart – yellow, fat – brown, liver – green.

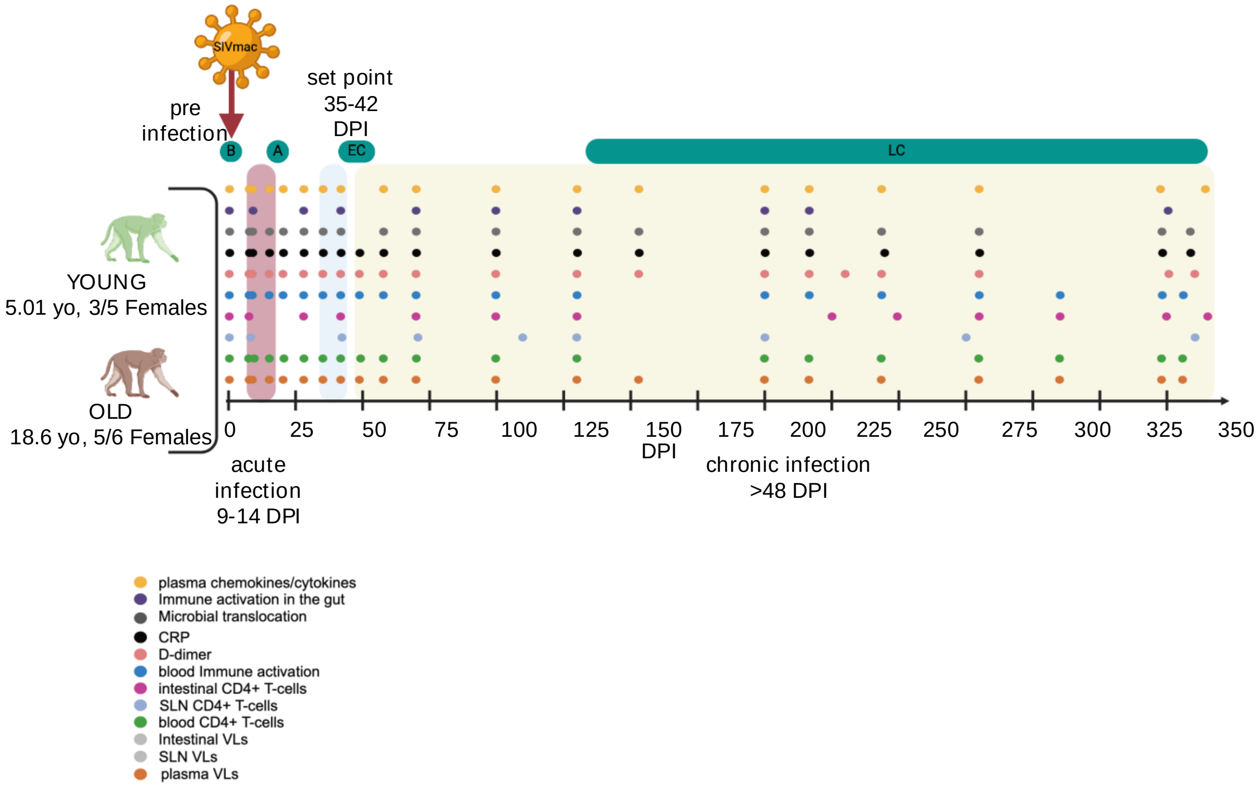

Supplemental Figure 13. Study design with highlighted most critical collection time points for biomarker analysis. The studies of the biomarkers of SIV infection included: viral loads in plasma, the superficial lymph nodes (LNs) and duodenum, immune activation and proliferation in the blood $CD4^+$ and $CD8^+$ T cells ($CD4^+CD67^+$, $CD8^+CD67^+$, $CD4^+CD25^+$, $CD8^+CD25^+$, $CD4^+CD38^+HLA\text{-}DR^+$, and $CD8^+CD38^+HLA\text{-}DR^+$ T cells), D-dimer in serum, CRP in plasma, microbial translocation (sCD163, sCD14) in plasma, immune activation in the gut ($CD4^+CD67^+$, $CD8^+CD67^+$, $CD4^+CD38^+HLA\text{-}DR^+$, and $CD8^+CD38^+HLA\text{-}DR^+$ T cells), chemokines/cytokines in plasma (IL-1B, IL-1RA, CXCL-9 (MIG), IL-15, IL-12, CXCL-11 (I-TAC), CXCL-8 (IL-8), and CXCL-10 (IP-10).
